# Supplementary material for: Malicious source code detection using a translation model
Source: Patterns (N Y). 2023 Jun 6;4(7):100773. doi: 10.1016/j.patter.2023.100773 (PMC10382987; doi:10.1016/j.patter.2023.100773)
Supplement: Document S1. Figures S1–S8 and Table S1 [file mmc1.pdf]

Patterns, Volume *4*

Supplemental information

Malicious source code detection using a translation model

Chen Tsfaty and Michael Fire

Supplemental information

All functions implementations Average Precision

Table S 1: *AP* for all of the functions in **??** with all attacks.

Function Name	Execution of an obfuscated string using ***exec***	Execution of a non obfuscated script using ***exec***	Execution of a obfuscated string using ***os.system***	Loading a file from the root directory of the program	Payload construction as an obfuscation use case
predict	0.954	0.942	0.951	0.94	0.944
decode	0.913	0.91	0.909	0.904	0.932
list	0.917	0.902	0.904	0.901	0.917
update	0.998	0.998	0.998	0.998	0.998
install	0.856	0.861	0.856	0.856	0.861
configure	0.858	0.86	0.878	0.878	0.878
run	0.994	0.994	0.994	0.995	0.994
post	0.914	0.905	0.903	0.906	0.906
encode	0.905	0.903	0.902	0.912	0.902
generate	0.773	0.773	0.8	0.773	0.775
draw	0.893	0.888	0.892	0.892	0.886
clean	0.999	0.999	0.999	0.999	0.999
set	0.993	0.99	0.99	0.992	0.99
data	0.912	0.913	0.914	0.909	0.908

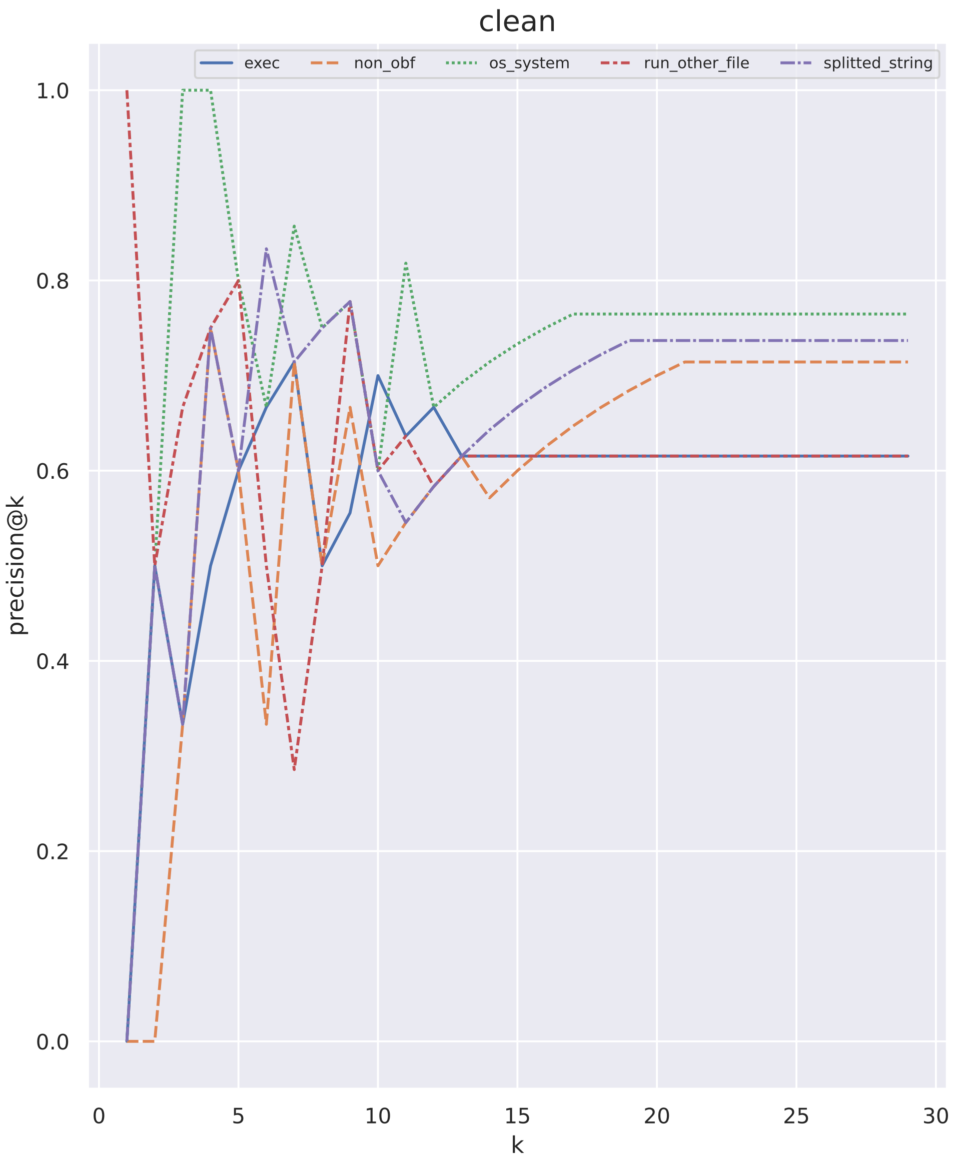

Figure S 1: The graphs below describe the *precision@k* results of the applied method in k in the range of 1 to 30. The presented results include all the *clean* function implementations with different attacks (with the random code injection)

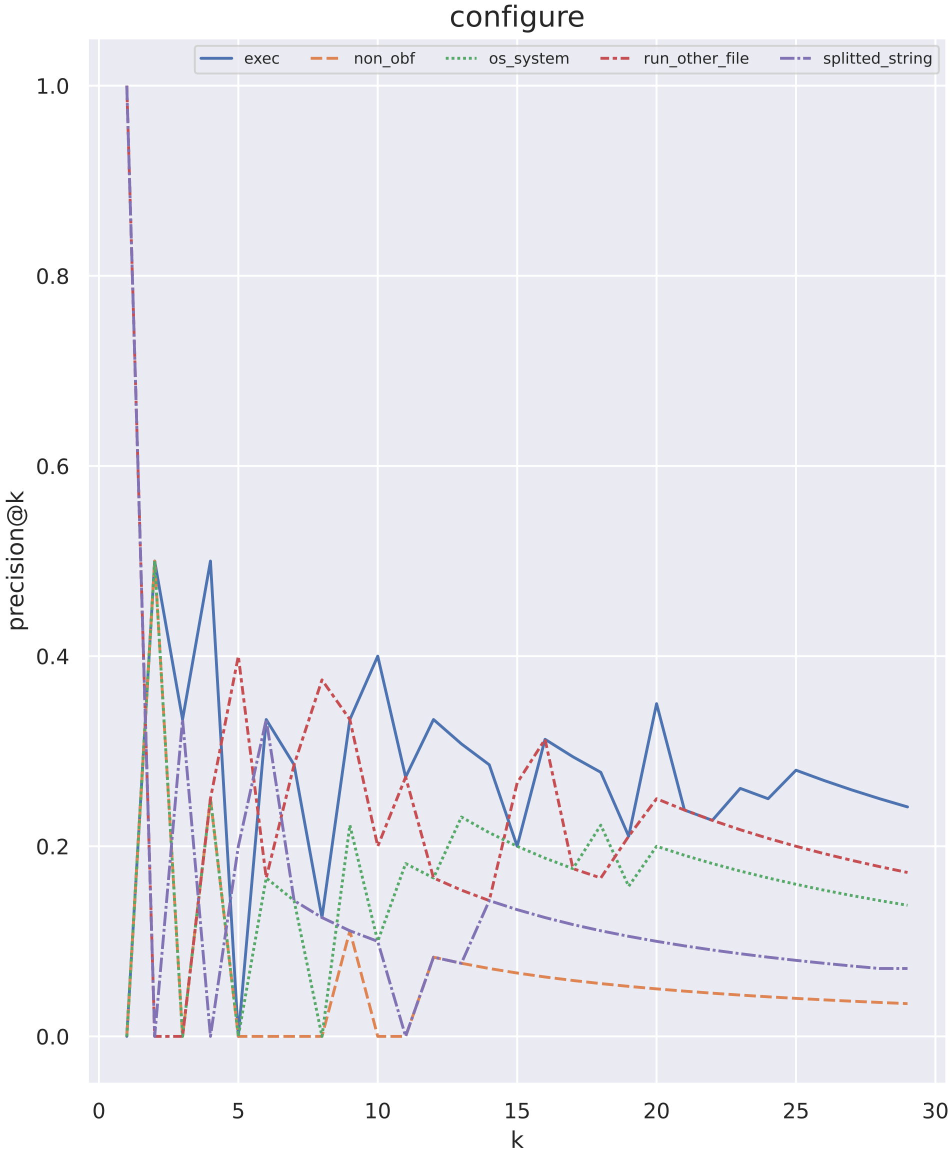

Figure S 2: The graphs below describe the *precision@k* results of the applied method in k in the range of 1 to 30. The presented results include all the *configure* function implementations with different attacks (with the random code injection)

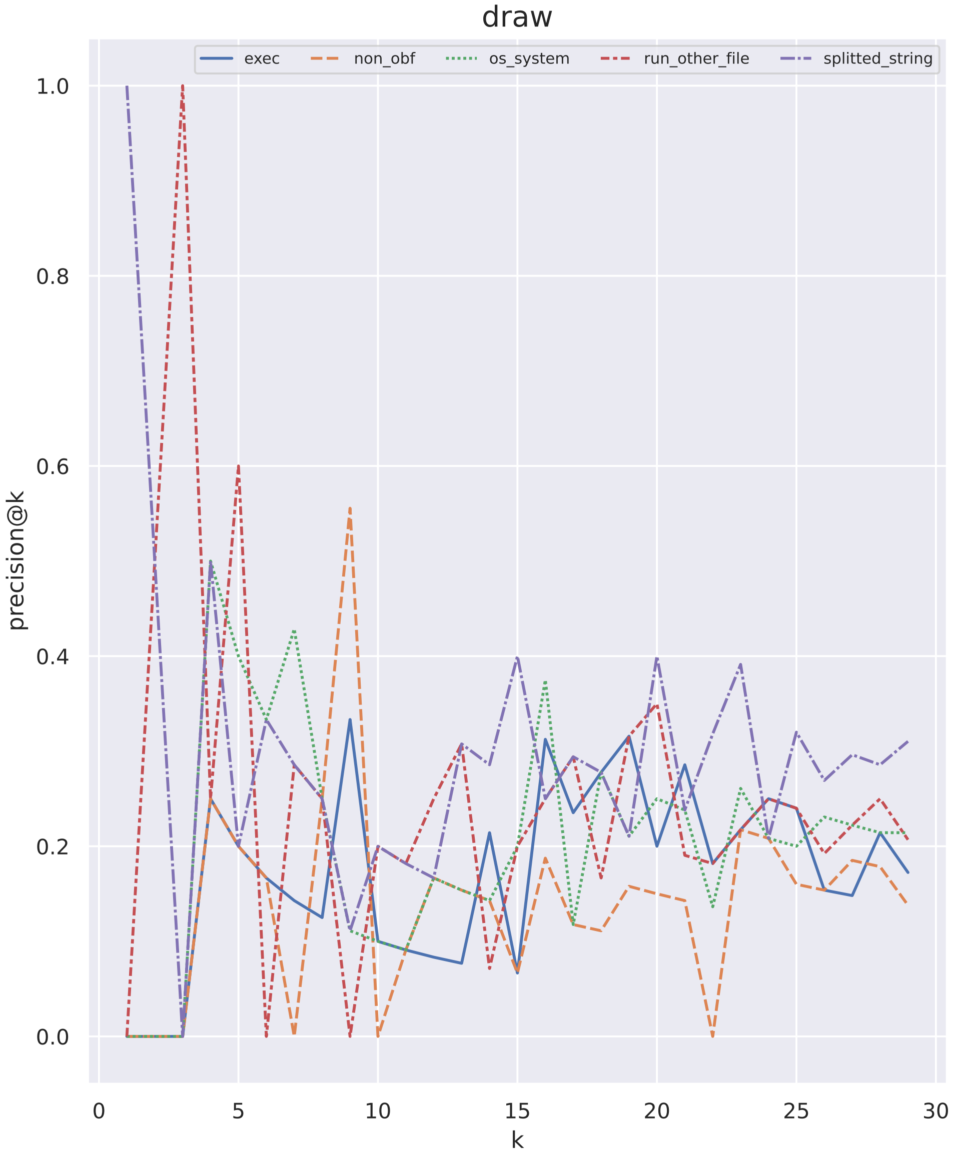

Figure S 3: The graphs below describe the *precision@k* results of the applied method in k in the range of 1 to 30. The presented results include all the *draw* function implementations with different attacks (with the random code injection)

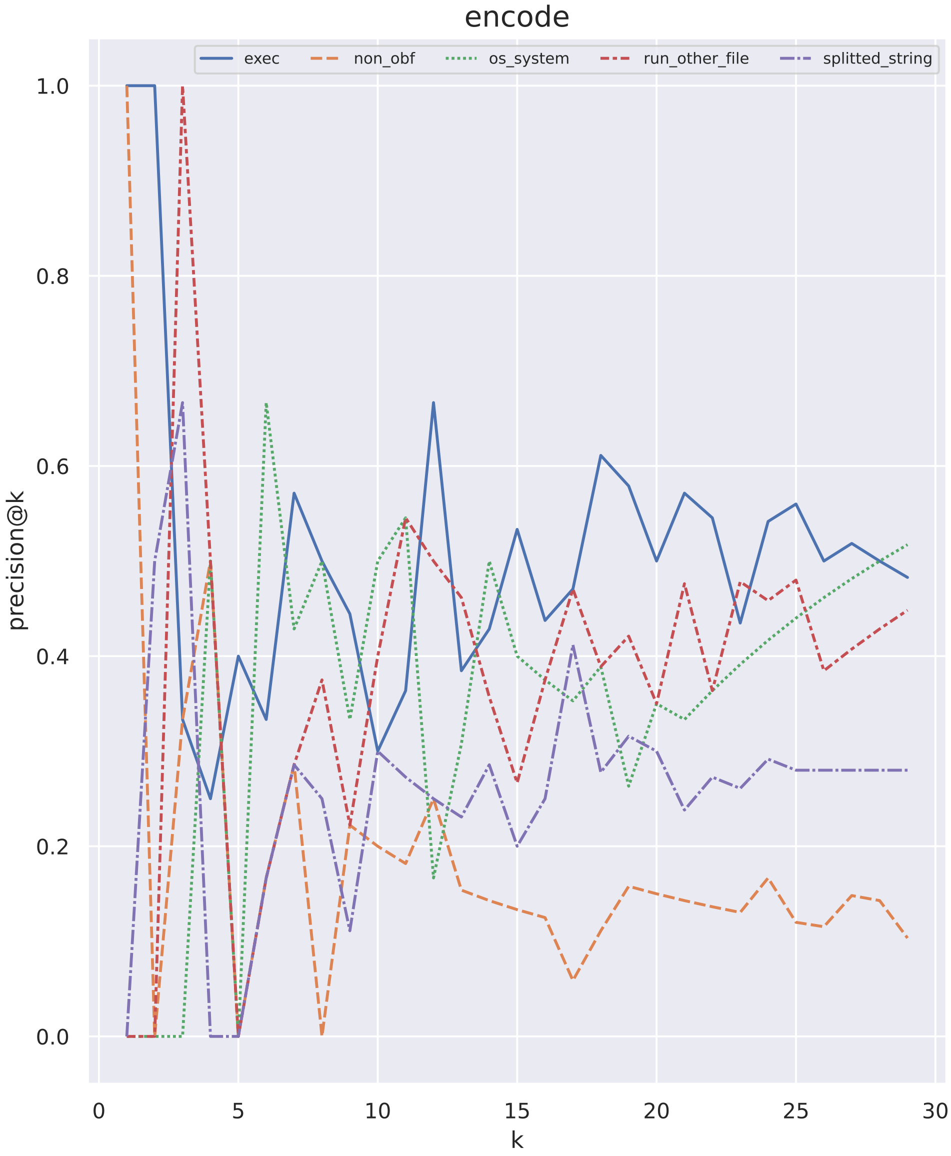

Figure S 4: The graphs below describe the *precision@k* results of the applied method in k in the range of 1 to 30. The presented results include all the *encode* function implementations with different attacks (with the random code injection)

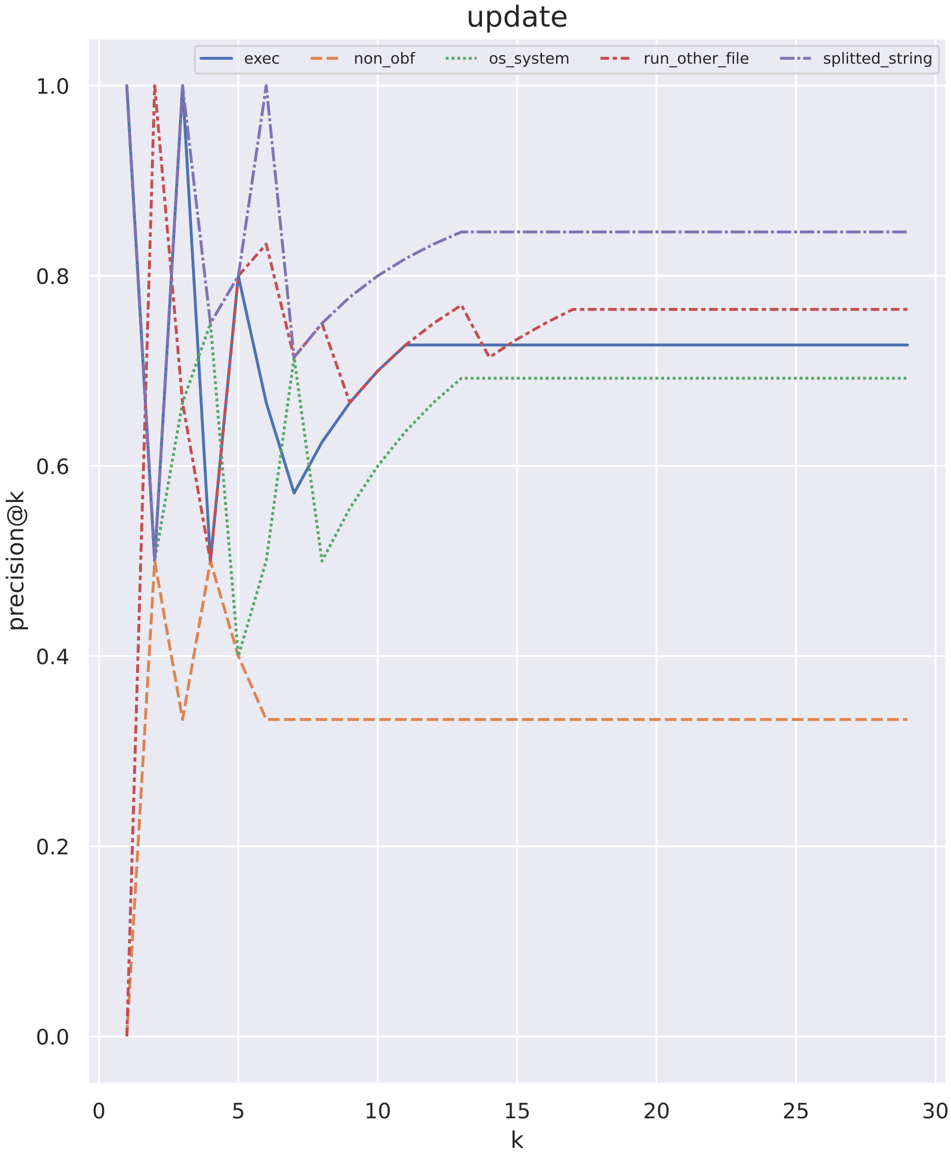

Figure S 5: The graphs below describe the *precision@k* results of the applied method in k in the range of 1 to 30. The presented results include all the *update* function implementations with different attacks (with the random code injection)

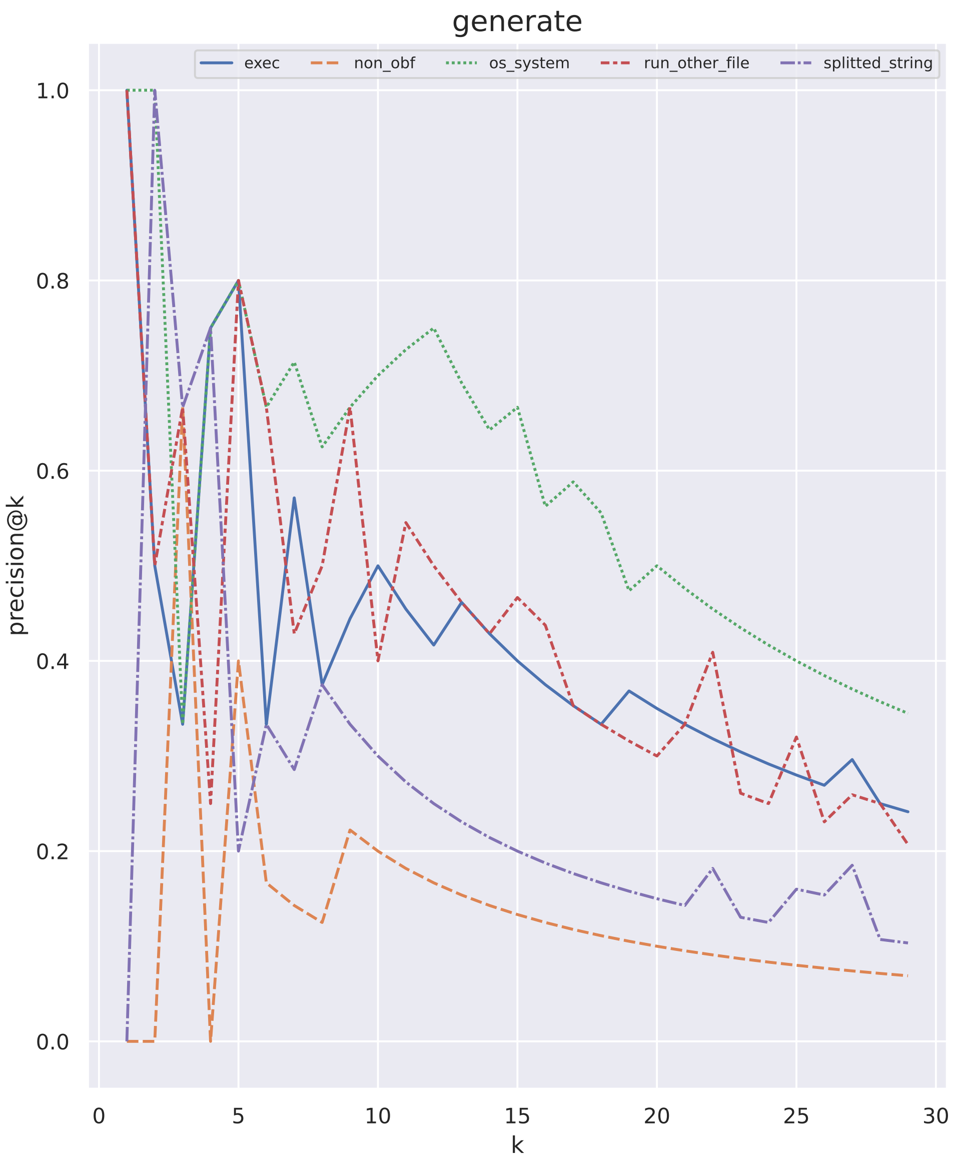

Figure S 6: The graphs below describe the *precision@k* results of the applied method in k in the range of 1 to 30. The presented results include all the *generate* function implementations with different attacks (with the random code injection)

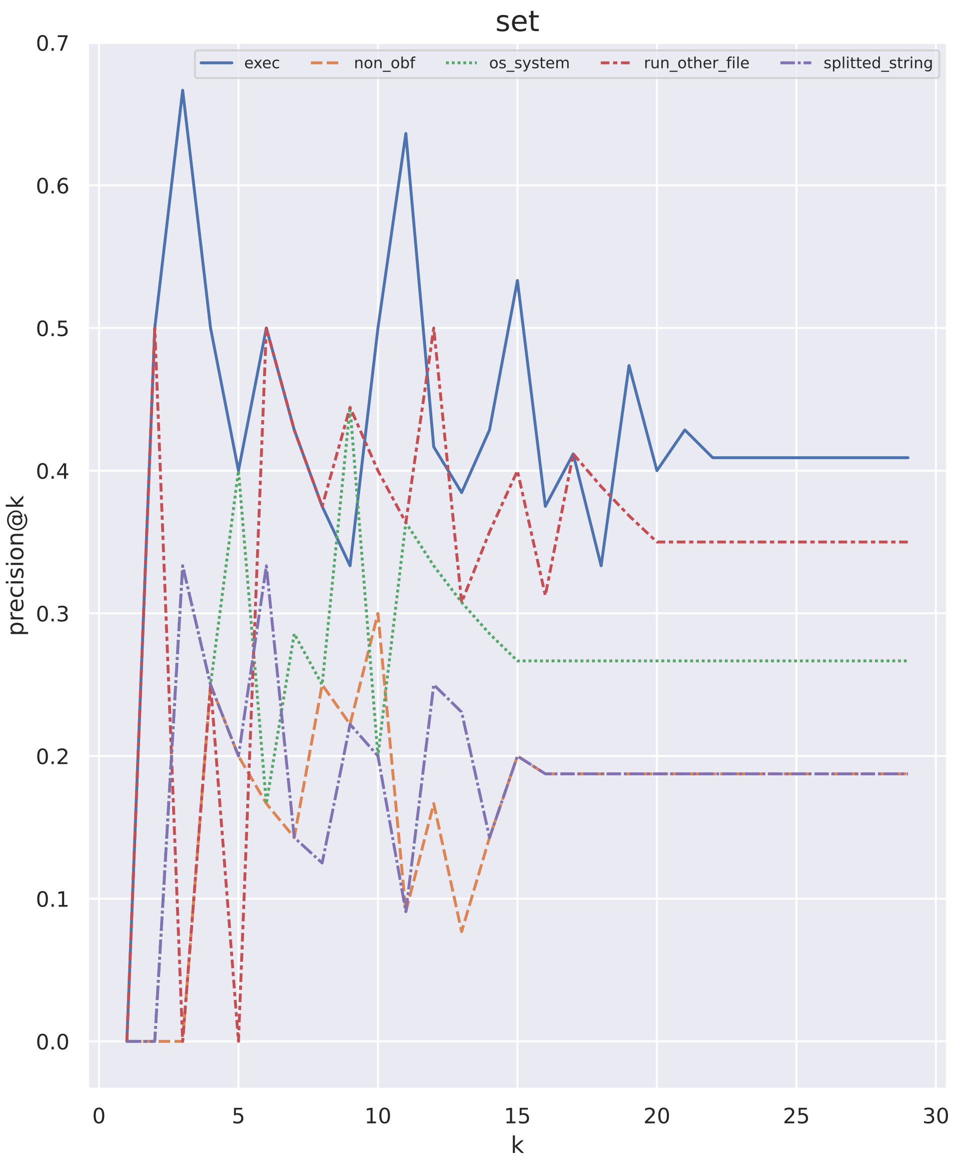

Figure S 7: The graphs below describe the *precision@k* results of the applied method in k in the range of 1 to 30. The presented results include all the *set* function implementations with different attacks (with the random code injection)

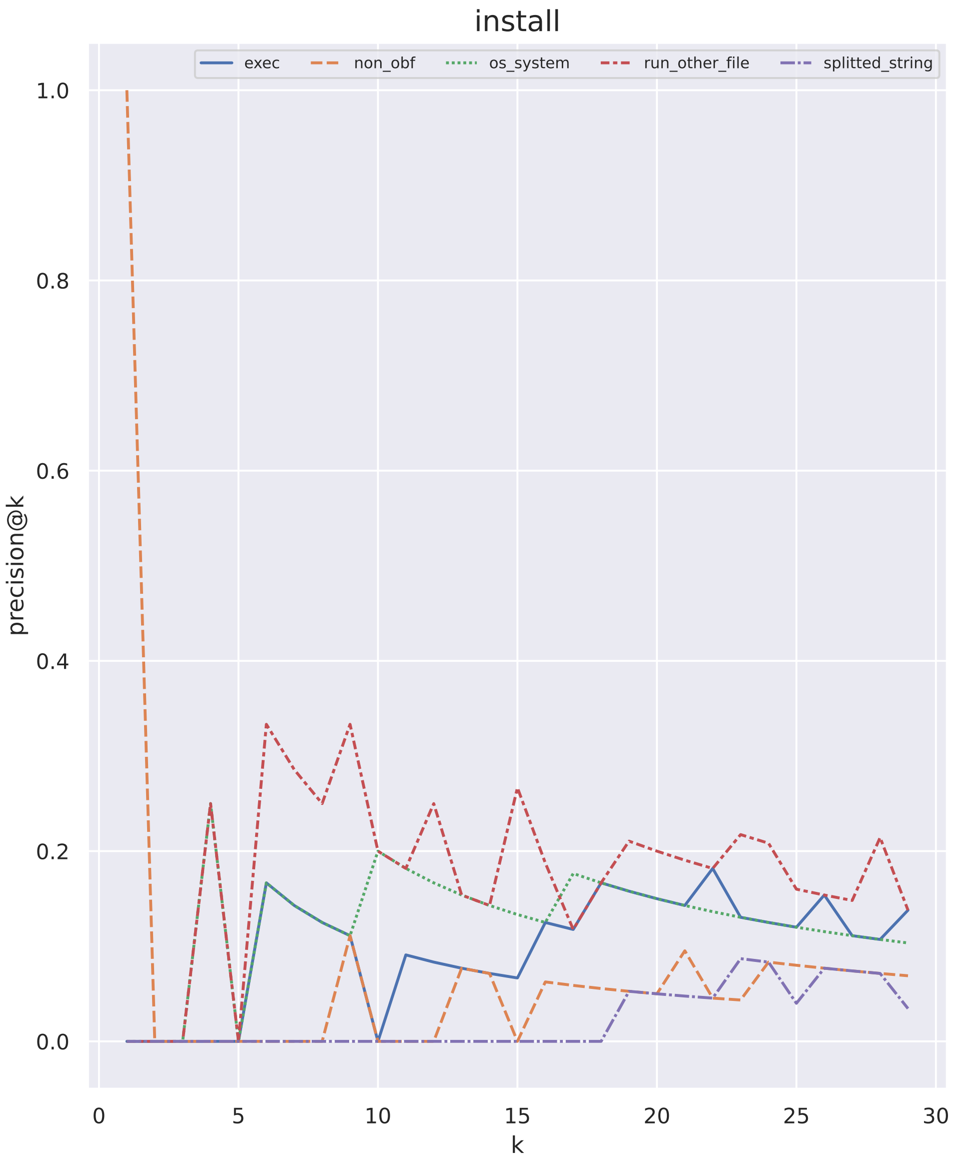

Figure S 8: The graphs below describe the *precision@k* results of the applied method in k in the range of 1 to 30. The presented results include all the *install* function implementations with different attacks (with the random code injection)
