# Supplementary material for: Malicious source code detection using a translation model
Source: Patterns (N Y). 2023 Jun 6;4(7):100773. doi: 10.1016/j.patter.2023.100773 (PMC10382987; doi:10.1016/j.patter.2023.100773)
Supplement: Document S2. Article plus supplemental information [file mmc2.pdf]

Article

Patterns

Malicious source code detection using a translation model

Highlights

- MSDT automatically detects code injection via anomaly detection in source code
- MSDT provides ranked anomalies measured by the *precision@k* metric
- A dataset of Python functions injected with real-world malicious codes is available
- With a given grammar, MSDT can support any programming language

Authors

Chen Tsfaty, Michael Fire

Correspondence

chents@post.bgu.ac.il (C.T.),
mickyfi@bgu.ac.il (M.F.)

In brief

Modern software development often includes open-source code. Broad communities are managing those codes; therefore, anyone can modify them. Recent years have seen hackers using this process to attack these communities and their users. MSDT, presented in this article, is an algorithm that detects malicious code injections at the source code level. It uses deep-learning and anomaly-detection practices applied to an open-source dataset of 607,461 functions, part of which were injected with several real-world malicious codes.

Tsfaty & Fire, 2023, Patterns *4*, 100773
July 14, 2023 © 2023 The Author(s).
https://doi.org/10.1016/j.patter.2023.100773

CellPress

Patterns

CellPress
OPEN ACCESS

Article

Malicious source code detection using a translation model

Chen Tsfaty[1,2,*] and Michael Fire[1,*]
[1]Department of Software and Information Systems Engineering, Ben-Gurion University, Beer-Sheva 8410501, Israel
[2]Lead contact
*Correspondence: chents@post.bgu.ac.il (C.T.), mickyfi@bgu.ac.il (M.F.)
https://doi.org/10.1016/j.patter.2023.100773

THE BIGGER PICTURE Most software development procedures today rely heavily on open-source codes managed in a community-based form so that anyone can contribute to their maintenance. The developers and their end users are targeted by bad actors using these properties to spread their malicious intentions. To address this challenge, we developed a method for detecting malicious intent in open-source code using deep-learning algorithms, an unsupervised method for discovering malicious codes. We demonstrate the method by detecting real-world malicious codes injected into randomly selected functions.
There are many opportunities for further experimental and computational studies of malware analysis and code security based on this proposed generic method, which lays out innovative principles for dealing with this type of threat.

SUMMARY

Modern software development often relies on open-source code sharing. Open-source code reuse, however, allows hackers to access wide developer communities, thereby potentially affecting many products. An increasing number of such "supply chain attacks" have occurred in recent years, taking advantage of open-source software development practices. Here, we introduce the Malicious Source code Detection using a Translation model (MSDT) algorithm. MSDT is a novel deep-learning-based analysis method that detects real-world code injections into source code packages. We have tested MSDT by embedding examples from a dataset of over 600,000 different functions and then applying a clustering algorithm to the resulting embedding vectors to identify malicious functions by detecting outliers. We evaluated MSDT's performance with extensive experiments and demonstrated that MSDT could detect malicious code injections with *precision@k* values of up to 0.909.

INTRODUCTION

Software supply chain attacks aim to access source code, build processes, or update mechanisms by infecting legitimate apps to distribute malware.[1] Hence, the end users will perceive that malware is safe and trustworthy software and will therefore be more likely to download it. An illustrative example is the Codecov attack,[2] where a backdoor concealed within a Codecov uploader script was widely downloaded. In April 2021, attackers compromised a Codecov server to inject malicious code into a bash uploader script. Codecov customers then downloaded this script for 2 months. When executed, the script exfiltrated sensitive information, including keys, tokens, and credentials from those customers' continuous integration/continuous delivery environments. Using these data, Codecov attackers reportedly breached hundreds of customer networks, including HashiCorp, Twilio, Rapid7, Monday.com, and e-commerce giant Mercari.[2]

These types of attacks are becoming increasingly popular and harmful due, in part, to modern development procedures that use open-source packages and public repositories.[3] These procedures are efficient, cost-effective, and accelerate development, and are therefore popular among many developers. There has been a 73% growth of open-source software component downloads in 2021 compared to 2020,[3] and a reported 77% increase in the use of open-source software between 2021 and 2022 among various companies.[4]

In addition, Red-Hat predicts an 8% decline in the use of proprietary software in software already in use in respondents' organizations over the next 2 years.[5] Over the same period, they expect enterprise open source to increase by 5% and community-based open source also to increase by 3% over the same period, resulting in open-source technologies being adopted more than any other technology. Development procedures involving those packages and repositories are mostly automatic,

© 2023 The Author(s).
This is an open access article under the CC BY license (http://creativecommons.org/licenses/by/4.0/).

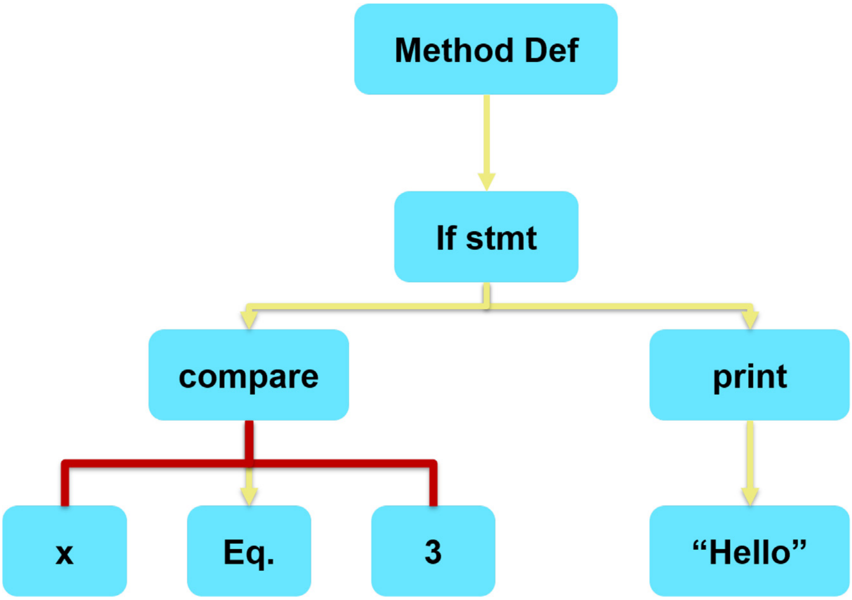

Figure 1. Example AST transformation of the code snippet if x = = 3: print("Hello")
Example of AST path shown in red.

or at least semi-automatic, the same as developers installing an open-source package.[6] As a result of this growth, popular packages, development communities, lead contributors, and many more can be considered attractive targets for software supply chain attacks.[7–11] These kinds of attacks can make dependent software projects more vulnerable. In 2021, OWASP (the Open Worldwide Application Security Project) considered software supply chain threats to be one of the top ten security issues worldwide. A lead example of such attacks was the *ua-parser-js* attack,[12] where in October 2021 the attacker was granted ownership of the package by account takeover and published three malicious versions. At that time, *ua-parser-js* was a highly popular package with more than 7 million weekly downloads.

In recent years, a vast research field has emerged to deal with this threat.[7,13,14] This field is researched by academia and is part of the application security market, which has been valued at US$6.2 billion.[15] This research field includes many aspects that depend on various parameters, such as programming language (PL). Different PLs have different security issues. For example, Python has assert statements that control the application logic or program execution, which can lead to the retrieval of incorrect results, introduce security risks, or cause program failure.[16] In C++, it is more common to commit buffer overruns by writing input to smaller buffers.[17] A second important parameter to consider is the scope of the functionalities being examined (e.g., function, class, scripts). For example, there are attacks targeting central locations in the package, such as the installation phase or fundamental functions.[18,19]

In this study, we developed the Malicious Source code Detection using a Translation model (MSDT) algorithm, a novel method for detecting malicious code injection within the functions' source code, by static analysis that consists of the following four key steps (see "the proposed method"). First, we used the *PY150* dataset[20] to train a deep neural architecture model. Second, by utilizing that model, we were able to embed every function in the CodeSearchNet (CSN) Python dataset, which is used for experimental evaluation,[21] into the representation space of the model's encoding part. Third, we applied a clustering algorithm over every function type implementation to detect anomalies by outlier research. Lastly, we ranked the anomalies by their distance from the nearest clusters' border points: the farther away from the point, the higher the score.

We conducted extensive experiments to evaluate MSDT's performance. We started by randomly injecting five different real-world malicious codes into the top 100 common functions, using Code2Seq[22] as the deep neural model and DBSCAN for the clustering algorithm.[23,24] Next, we measured the precision at k (*precision@k*) (for various k values) of MSDT's ability to match functions classified as malicious with their proper tagging (see "experiments"). The *precision@k* test result values were as high as 0.909. For example, MSDT achieved this result when $k = 20$ for the different implementations of the *get* function. These implementations were randomly injected as part of a real-world attack described by Bertus.[18] In addition, we empirically evaluated MSDT on a real-world attack and succeeded in detecting it. Lastly, we empirically compared MSDT against widely used static analysis tools, which can only work on files. As MSDT works on functions, it has a more precise capability to detect an injection in a given function.

In addition to the MSDT algorithm itself, we also described and shared our open, curated dataset of 607,461 functions, some of which were injected with several real-world malicious codes in this work. This dataset can be used in future works within the field of code-injection detection.

Background

Malformed open-source packages constitute several threats to every component in some development procedures. Research within the vast field has three main branches.[25] In the following subsections, we provide an overview of these strands: the first section introduces an overview of the security issues that commonly appear in public repositories or occur because the PL features weaknesses exploitation. The second section provides an overview of widely used methods to detect those attacks or weaknesses. The third section presents an overview of the different deep-learning methods in the field of code representation, which are used to apply advanced static analysis to the targeted code.

Open-source packages' security issues

In recent years, the awareness of the threats regarding public repositories and open-source packages has increased. As a result, many studies[13,26,27] point out two main security issues with the use of such packages: (1) vulnerable packages[28] and (2) malicious intent in packages.[29] Vulnerable packages contain a flaw in their

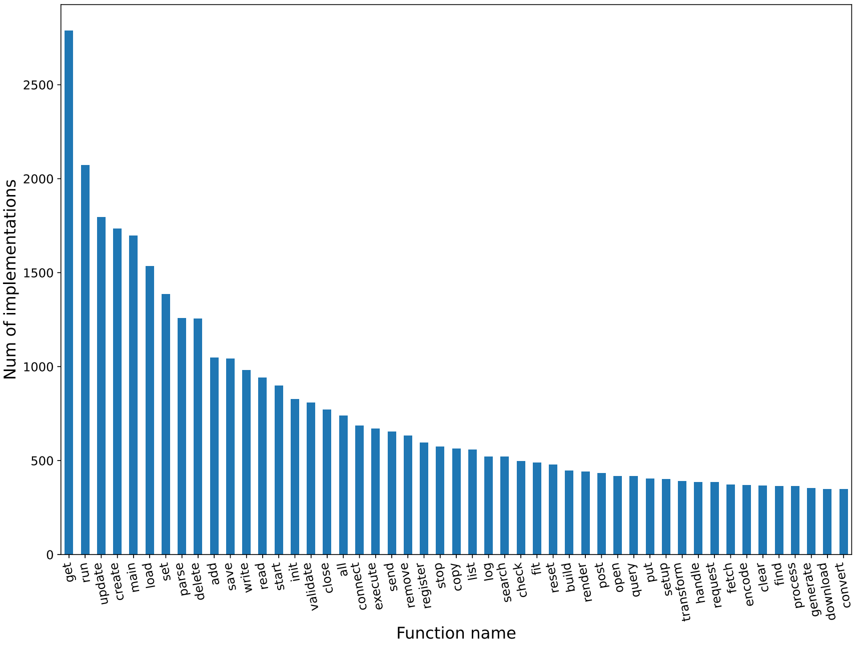

Figure 2. Number of different implementations per function type

design,[30] unhandled code error,[31] or other bad practices that could be a future security risk.[32,33] Communities and commercial companies have vastly researched this widespread threat (e.g., Snyk and Mend). Usually this threat is based on common vulnerabilities and exposures (CVEs). Those vulnerabilities allow the malicious actor, with prior knowledge of the package usage location, to achieve its goal with a few actions.[34,35] Malicious intent in packages[29] includes bad design, unhandled code error, or a code that does not serve the main functionality of the program. These examples are created to be exploited or triggered during some phases of the package (e.g., installation, test, runtime).

Studies have shown a rise in malicious functionalities appearing in public repositories and highly used packages.[32,36,37] These studies have shown that there are common injection methods for malicious actors to infect packages. As Ohm et al.[13] demonstrated, to inject malicious code into a package, an attacker may either infect an existing package or create a new one similar to the original one (which is often called dependency confusion[27]). A new malicious package developed and published by a malicious actor has to follow several principles: (1) for a proper replacement to be made to the targeted package, it has to contain a proper replacement to the targeted package a semi-ident functionality; and (2) it has to be attractive, ending up in the targeted users' dependency tree. To grant the use of the new package types, one of the following methods can be employed: naming the malicious package in a similar manner to the original one (typosquatting)[18,27,29,38]; creating a trojan in the package[19,39]; or using an unmaintained package or user account (use after free).[40] The second injection strategy can infect existing packages through one of the following methods: (1) injection to the source of the original package by a Pull request/social engineering[6,41–43]; (2) the open-source project owner adding malicious functionality out of ideology, such as political[44]; (3) injection during the build process[45]; and (4) injection through the repositories system.[46]

Ohm et al.[13] demonstrated that the malicious intent in packages could be categorized by several parameters: targeted operating system (OS), PL, the actual malicious activity, and the location of the malicious functionality within the package (where it is injected), among others. Additionally they showed that the majority of the maliciousness is associated with persistence purposes, which can be categorized into several major groups: backdoors, droppers, and data exfiltration.[13]

This study focuses on the second security issue with a specification in a dynamic PL (with Python as a test case) for usage popularity and the popularity of injection-oriented attacks within those PL repositories (e.g., Node.js, Python).[13] These injections are often related to the PLs' dynamicity features,[16] such as exposing the running functionalities only at runtime (e.g., exec("print (Hello world!)")), configurable dependencies, and imports of packages (e.g., import from a local package instead of a global one).

The described use of the PLs' dynamicity features is the most common among the known attacks.[13,47] A leading example of this kind of attack was presented by Bertus,[18] who reviewed a malicious package named "pytz3-dev," which was seen in the central repository of Python packages, the Python package index (PyPi), and downloaded by many. This package contains malicious code in the initialization module and searches for a Discord authentication token stored in an SQLite database. If found, the code exfiltrated the token. This attack was carried out unnoticed for 7 months and downloaded by 3,000 users in 3 months.[18,47] These features, and many more, are used by attackers, thus making it one of the most common attack techniques associated with a supply chain attack, as covered by the National Institute of Standards and Technology.[7]

Detection methods of malicious intent in source code

As a result of the increase in the security issues mentioned above, two primary detection methods were developed.

Static analysis. Static analysis finds irregularities in a program without executing it. The irregularities can broadly be categorized into three main branches: coding style enforcement, reliability, and maintainability.[32,48] The security issues are mainly associated with the reliability domain, covering bug detection,[49] vulnerability detection,[50] and malware detection challenges.[51,52] In static analysis, the following techniques are commonly used to gather information regarding the detection mission.

- Syntax properties uses the PL syntax to find irregularities. For example, abstract syntax tree (AST)[53] is a well-known data structure for representing a program with a given PL grammar and can be used to search the obfuscated strings most likely to be executed,[54] finding some similarity to

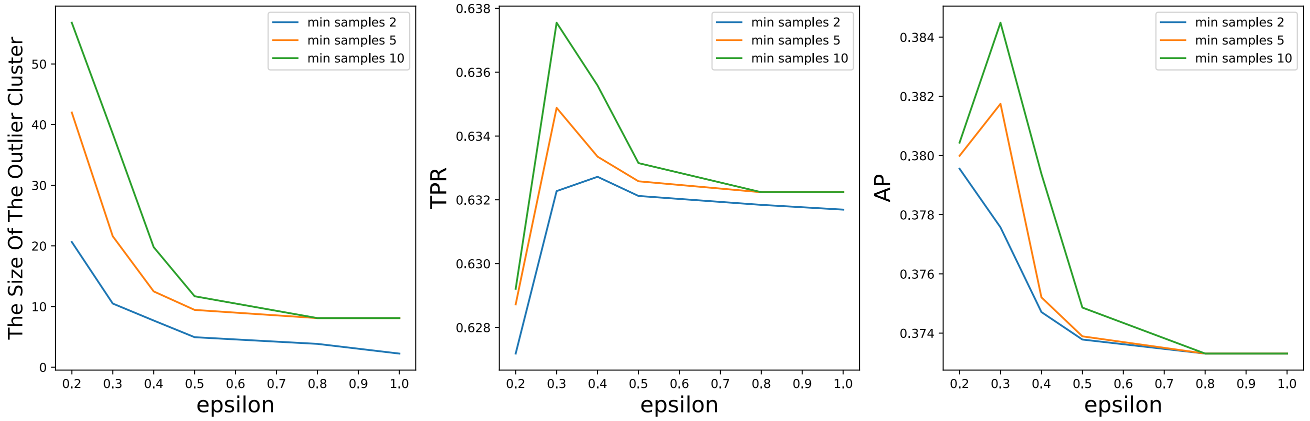

Figure 3. DBSCAN parameter tuning with TPR

The graphs from left to right show the DBSCAN parameter tuning process: (1) the size of the outlier cluster, which indicates whether the methods overfit or underfit; (2) the measured *precision@k* for a range of *k*; and (3) the measured AP for a range of *k*.

malware by code similarity techniques,[55] or a *linter* operation to check the program's correctness.[48]

- Feature-based technique uses the occurrences count of known problematic functionalities.[32,56] For example, Patil and Patil[52] have constructed a classifier with a given labeled dataset and several features extracted (such as function appearances, length of the script) that can predict the maliciousness of a script. The main drawback of this technique is that it strongly binds with reversing research that points to features related to the attack, which may lead to detection overfitting the attacks that have been revealed and learned. Furthermore, potential attackers could evade detection by several methods, such as not using or not adequately using the searched features in the code.[57] An example of such a static analysis tool is Bandit.[58] Bandit is a widespread tool[32] designed to find common security issues in Python files using hard-coded rules. This tool uses the AST (see "deep-learning methods for analyzing source code") form of the source code to better examine the rule set. In addition, Bandit's detection method includes the following metrics: severity of the issues detected and the confidence of detection for a given issue. Those metrics are divided into three values: low, medium, and high. Each rule manually obtains its severity and confidence values from the Bandit community.
- Data preprocess constructs a workable data structure that grasps the code's syntax and semantic information to better represent it (see "deep-learning methods for analyzing source code"). The anomaly detection or classification research can be conveniently applied with a proper code representation. For example, Alomari and Harbi[59] constructed a control flow graph that identified similar code segments between programs by using resemblance subgraphs.
- Signature-based detection (in the case of malware detection) is a process whereby a set of rules (based on reversing procedure) define the maliciousness level of the program.[60] Rules generated for static analysis purposes are often a set of functionalities or opcodes in a specific order to match the researched code behavior. For example, YARA is a commonly used static signature tool, and the generated rules for dynamic analysis purposes are often a set of executed operations, memory states, and registers' values.[51,61] The main drawback of this technique is that it applies to known maliciousness.
- Comparing packages with known CVEs (see "open-source packages' security issues").

On the one hand, static analysis tends to scale well over many PL classes (with a given grammar), efficiently operating on large corpora. It often identifies well-known security issues and in many cases is explainable.[62] On the other hand, this kind of analysis suffers from a high number of false positives and poor detection of configuration issues.[63]

Dynamic analysis. This type of analysis finds irregularities in a program after its execution and determines its maliciousness, where gathered data, such as system calls, variable values, and IO access are often used for anomaly detection or classification problems.[51] There are several drawbacks to using dynamic analysis on a source code[64]: (1) data-gathering difficulties: the procedure of extracting data is difficult to automate, as the package needs to be activated and its functionality executed; (2) scalability: the learned and tested program must be activated in its entirety, whereby the desired data have to be extracted for each. Therefore, in this study we have chosen to focus on advanced static analysis.

Deep-learning methods for analyzing source code

In recent years, there has been an increasing need to use machine learning (ML) methods in code intelligence for productivity and security improvement.[65] As a result, many studies construct statistical models to code intelligence tasks. Recently, pretrained models were constructed by learning from big PL corpora, such as CodeBERT[66] and CodeX.[67] These pretrained models are commonly based on models from the natural language process field (such as BERT[68] and GPT[69]), including improvements of the original Transformer architecture and the original self-attention mechanisms presented by Vaswani et al.[70] Not only did this development lead to improvement in code understanding[65] and generation problems,[71] but it also enlarged the number of tasks and their necessity,[65] such as clone detection[72]

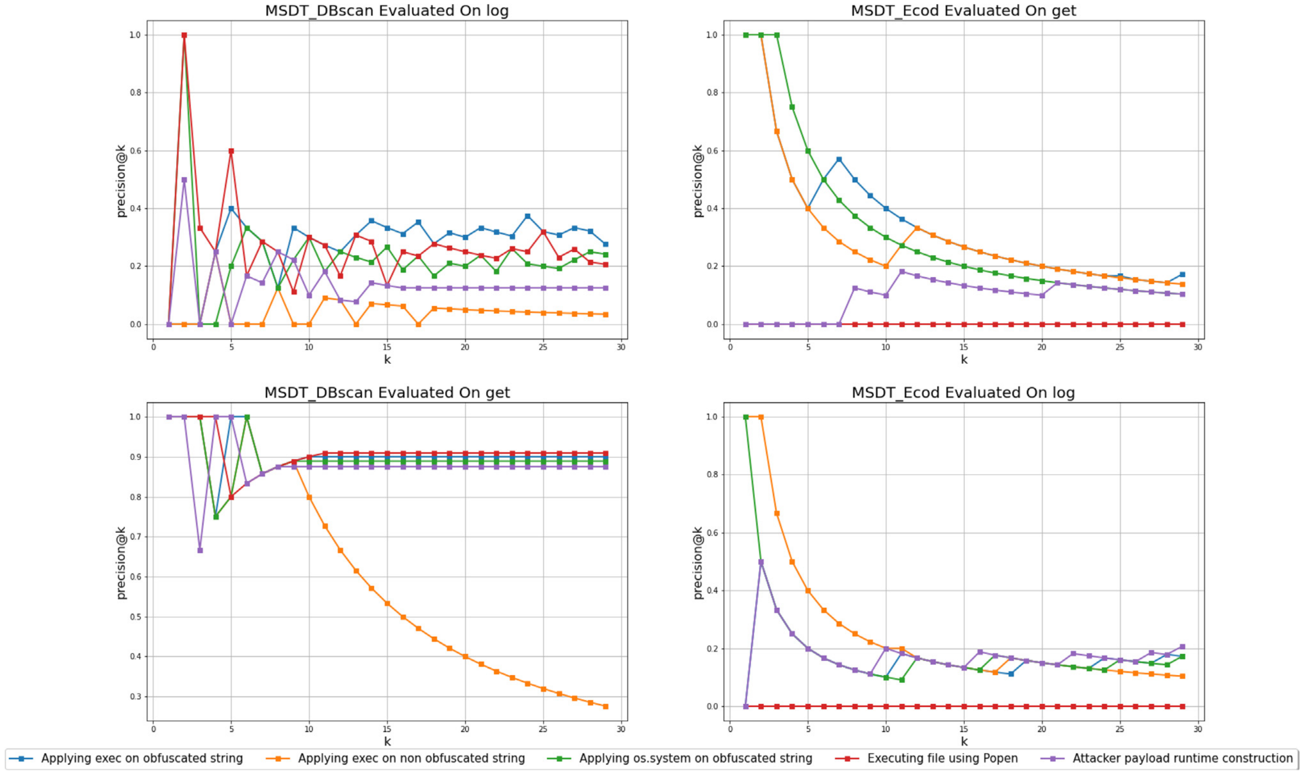

Figure 4. The measured *precision@k* of $MSDT_{DBSCAN}$ and $MSDT_{Ecod}$ of the *get* and the *log* functions' implementations

and code completion.[73] Those tasks include several challenges, such as capturing semantic essence,[74] syntax resemblance,[59] and figure execution flow.[75] For every challenge, it occurred that a model exists that would fit better than others.[65] For example, for code translating between PLs, algorithms including a "cross-lingual language model" with masked token preprocessing are superior for capturing well the semantic essence.[66,76]

Over the years, several ML methods have been researched within the context of code analysis tasks. In 2012, Hovsepyan et al.[77] showed the use of techniques from the classic text analysis field, for example using support vector machines algorithm on a bag-of-words representation of simple tokenization (lexing by the PL grammar) of Java source. In 2016, Dam et al.[78] and Liang and Zhu[79] presented techniques to obtain context for the extracted tokens using, for example, the output of recurrent neural network (RNN) trained over tokenized (lexing representations) code.[78] However, Ahmad et al.[80] showed that RNN-based sequence models lack several source code concepts regarding source code representations: first, inaccurate representation of the non-sequential structure of source code; second, RNN-based models may be inefficient for very long sequences; third, those models lack the ability to grasp the syntactic and semantic information of the source code. Therefore, starting in 2018, studies included two significant learning source code representation changes. The first of these changes is the use of Transformers, which has proven efficient in capturing long-range dependencies.[71] The second change is the use of different data preprocessing procedures, which yield more informative data structures on which to learn: indeed, Alon et al.[22] used AST paths for a deep neural architecture named Code2Seq,[22] and Mou et al.[81] utilized AST nodes to train tree-based convolutional neural networks for supervised classification problems. Moreover, AST enables reliable source code preprocessing, resulting in an object containing information about the program's structure and allowing for extracting program execution information.[14] Lately, researchers have tried to include semantic data of the PLs. For example, Feng et al.[66] presented the CodeBERT model, which uses a bimodal pretrained model to learn the semantic relationship between natural languages and PLs, such as Java, PHP, and Python.

In this study we used the Code2Seq model, which is a deep neural architecture developed by Alon et al.[22] We selected this model over others because it performs the mentioned code-embedding models in a similar task, such as Code Search, and Code Captioning.[22,82] Additionally, as Han et al.[82] demonstrated, the Code2Seq model has fewer parameters compared to other models. Similarly to Ramakrishnan et al.,[83] we trained the model using the PY150 dataset.[81] This dataset contains Python functions in the form of AST (see "datasets"). In this architecture, a function is referred to as an AST where the output trees' internal nodes represent the program's construction with known rules, as described in the given grammar. The tree's leaves represent information regarding the program variables, such as names, types, and values. Figure 1 outlines the notion of AST on code snippets. Eventually, the Code2Seq model obtains a set of AST paths, where every pairwise path between two leaf tokens is represented as a sequence containing the

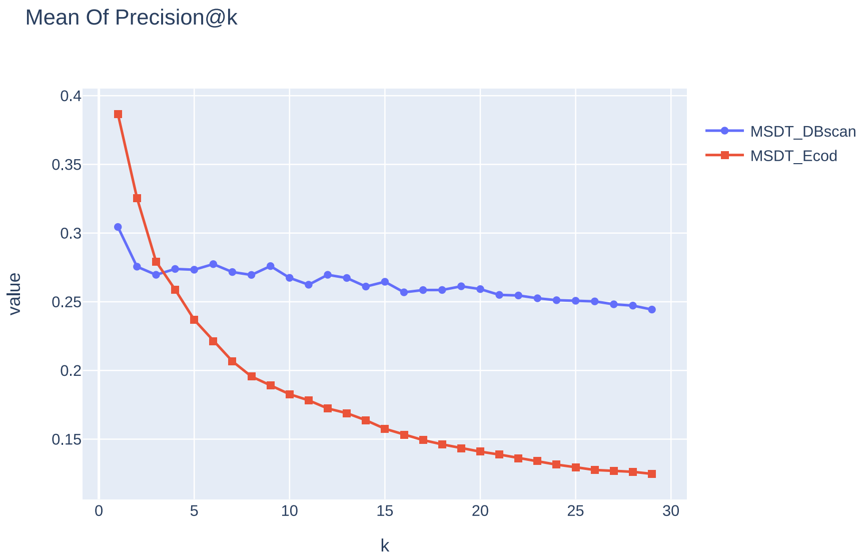

Figure 5. The measured mean *precision@k* of $MSDT_{DBSCAN}$ and $MSDT_{Ecod}$ of all the 100 function types and the five attacks

AST nodes. Up and down arrows connect those nodes, exemplifying the up- or downlink between the nodes in the tree. An example of an AST path is shown in Figure 1: (x, ↑if stmt, ↑method dec ↓print: "Hello"), extracted from code snippets as input. A bidirectional long short-term memory (LSTM) then encodes those paths, creating a separate vector representation for each path and its AST values. Next, the decoder attends to the encoded paths while generating the target sequence. The final output of the Code2Seq model generates a sequence of words that explain the functionality of the given code snippet.[22] For example, with a source code function of calculation power of 2 of a given variable that inputted to the Code2Seq model, the result was in an output word sequence of "Get Power Of Two."

Code2Seq can be integrated into many applications,[22,74,83] such as Code Search, with a given sentence describing a code, and the output will be the desired code. For example, Nagar[74] used the Code2Seq model to generate comments for collected code snippets. The candidate code snippets and corresponding machine-generated comments were stored in a database where, eventually, the code snippets with similar comments to natural language queries were retrieved.

Recent studies have presented more advanced code-embedding methods that aim to include the program's semantic, syntactic, and execution flow as part of the representation.[59,75]

RESULTS

This section presents the experimental results obtained by the MSDT algorithm (see "the proposed method") when applied to the constructed function types dataset that contained both injected and benign implementations (see "injection simulation"). It is worth noting that this study used an 8 GB RAM with 8 CPU cores server to evaluate the algorithm. The runtime of the process took about 10 min for 48,627 different implementations.

The constructed dataset includes the 100 most common function types from the CSN dataset (see "datasets"). From the function type implementations distribution (Figure 2), the most common function type is the *get* function with over 3,000 unique implementations, and the least common is the *prepare* function with 102 unique implementations.

The first experiment included parameter tuning of the DBSCAN method mentioned in "anomaly detection on representation," which we applied to the CSN dataset without the 100 most common function types. We received the following best results (Figure 3) for $eps = 0.3$ and $min\_samples = 10$: $TPR = 0.637, AP = 0.384$ and $outlier\_detection\_precision = 0.953$. These results indicate that it is possible to detect anomalies by finding outliers with probable rates. Furthermore, when the default values of the DBSCAN method were set,[84] it obtained $TPR = 0.632$, $AP = 0.373$, and $outlier\_detection\_precision = 0.738$. Therefore, the DBSCAN with the tuned parameters exceeded the one with the default parameters.

The second experiment included the evaluation of $MSDT_{DBSCAN}$ on every function type against every attack type and every k in the range of 1%–10% of the implementations. For every iteration of k, we measured *precision@k*. We found that $MSDT_{DBSCAN}$ detects well when applied to several functions and attacks. For example, for the *get* function with three of the mentioned attacks, for $k = 10$ MSDT presented the highest value of $precision@10 = 0.909$ (Figure 4), compared with $precision@10 = 0$, which the *RandomClassifier* obtained. On the other hand, we found that $MSDT_{DBSCAN}$ achieved less successful results on several functions, no matter the type of the applied attack and the value of k, such as the *log* function with all the attacks, specifically the non-obfuscated attack. Table S1 and Figure S1 present in detail the results of these experiments, where the average precision (AP) of these experiments are shown to demonstrate the complete picture of the classification's nature.

In addition, we discovered that the measured Spearman's rank correlation between the MSDT'S detection rate and the number of implementations is equal to $\rho = 0.539$, indicating a correlation between the detection rate and the number of implementations. We also tested the $MSDT_{Ecod}$ on the same experimental settings described in "Code2Seq representation." Following the mentioned evaluation (see "evaluation process"), we measured the *precision@k* for every k ranging from 1 to 30. We can observe that generally, the $MSDT_{Ecod}$ detects the top two ranked anomalies and is less successful in the following k values (Figure 5).

The third experiment included detecting injected malicious implementations of *multiply* by applying $MSDT_{DBSCAN}$. By visualizing the principal component analysis (PCA; two components) of the collected samples (Figure 6), we can see that detecting the attacked functions, in this case, is a complex task. Additionally we can see (Figure 6) that by applying $MSDT_{DBSCAN}$, we

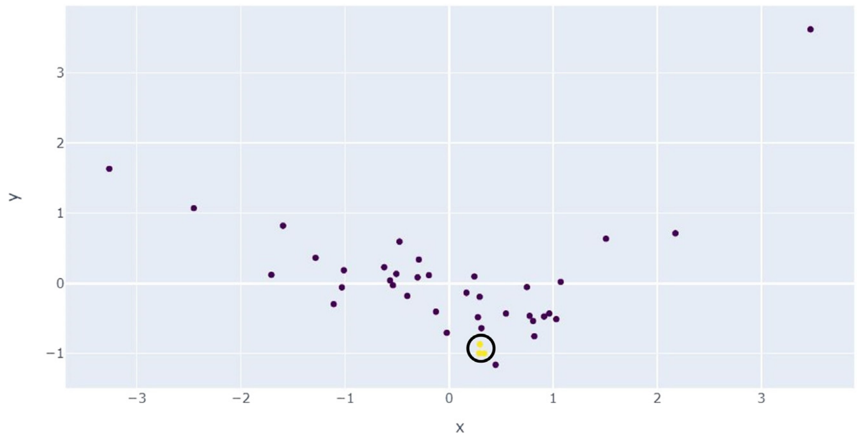

Figure 6. PCA (two components) visualization of real-case detection

The red data point is the attacked function, and the two yellow data points are the unique functions.

managed to detect the malicious implementation, along with two unique and odd implementations. Those implementations include: (1) adding in a loop for the first input number by the second input number; and (2) outputting the result by comparing the two input numbers with a results dictionary. We then compared the results of this experiment with Bandit and Snyk, yielding that the static analysis tools failed to detect these attacks. Additionally we compared $MSDT_{DBSCAN}$ with $MSDT_{Ecod}$, which detects only one of the mentioned unique implementations.

The fourth experiment emphasizes the relations between malicious and benign implementations. By the following visualization, we discerned (Figure 7) that the *get* functions tend to cluster while *log* functions do not cluster well. Therefore, this illustrates the differences in the distribution of the various function types.

DISCUSSION

Based on our analysis of the results presented in the results section and Figures S1–S8, we can observe the following.

First, $MSDT_{DBSCAN}$, which detects malicious code injections to functions by anomaly detection on an embedding layer, had promising results when evaluated on different function types with various injected attacks, reaching *precision@k* up to 0.909 with median = 0.889 and mean = 0.807 for *get* and *list* function types (Figures S1 and 4).

Second, $MSDT_{DBSCAN}$ succeeded in comparison with other tools and methods (Table 1 and Figure 5). For example, the general *precision@k* of $MSDT_{DBSCAN}$ is higher for $k>2$ compared with the $MSDT_{Ecod}$-based method (Figure 5). As mentioned in "injection simulation," the simulated injections are taken from real-world cases and injected into functions. To illustrate real-world code-injection detection we conducted an empirical experiment, which includes detecting real-world attacks by $MSDT_{DBSCAN}$ (see "evaluation process"). $MSDT_{DBSCAN}$ results seem promising compared to other widely used static analysis tools and $MSDT_{Ecod}$ in this specific case (Figure 6 and results). In the future, we plan to evaluate $MSDT_{DBSCAN}$ on other real-world cases and test it on different PL functions. It is also worth noting that the mentioned static analysis tools can only work on files, while MSDT works on functions. While this gives a more precise ability to detect code injections in functions, when applied to rare functions without many implementations MSDT would not necessarily succeed. In this case, we would like to test whether using MSDT on similar functions helps to detect code injection in rare functions.

Third, we observed similar results when $MSDT_{DBSCAN}$ evaluated similar attacks—for example, the attacks that utilized *exec* and *os.system* (as seen in *get* results in Figure 4) using the same payload but different execution functions. Additionally, we can see that the *precision@k* values are relatively similar for these two attacks in general (Figure S1). This conclusion shows us that if $MSDT_{DBSCAN}$ manages to detect one attack well, it should detect another semantically related attack, which should be explored further in future works.

Fourth, we found that $MSDT_{DBSCAN}$ seems to succeed when applied to functions with specific functionality that repeat in the various implementations of the same function type. For example, the *update* implementations tend to be similar—in general, this type of function gets an object and calculates or gets as an input a new value to insert in the given object—as we can see in Figure S1 for functions such as *list* and *update* with the main functionality and a relatively high *precision@k*. In this case, the various implementations of the same function type are semantically similar, yielding that the embedding for each is close and hence clusters well (see Figure 7 for illustration).

Fifth, we found that $MSDT_{DBSCAN}$'s detection rate positively correlates with the number of implementations in the function type. Hence, $MSDT_{DBSCAN}$ is more likely to achieve a higher detection rate with a more common function type with numerous implementations.

Sixth, when injecting attacks with extensive line lengths, such as the non-obfuscated script execution, $MSDT_{DBSCAN}$ tends to achieve less successful results (Figure 4). For example, when evaluating $MSDT_{DBSCAN}$ on the different function types injected with the non-obfuscated script, we generally obtain a low *precision@k* (Figure S1). In this case, the injected functionality is a script with numerous lines, which probably affects the Code2Seq robustness and causes it to mis-infer the function's functionality, as research by Ramakrishnan et al. shows.[83] In future work, we would like to create with Code2Seq a more robust model for source code (such as Seq2Seq[83]), a stacking model to overcome Code2Seq vulnerabilities.

Seventh, we can observe that $MSDT_{DBSCAN}$ tended to achieve less successful results when applied to abstract functions with functionality that does not repeat in other implementations for functions such as *run* and *configure*, as we can see in Figure S1. For example, the *install* function generally is supposed to change the state of the endpoint by activities that belong to the installation process (each application has a different process), such as writing files to disk or establishing a connection with a remote server. Each application has a different process with its unique

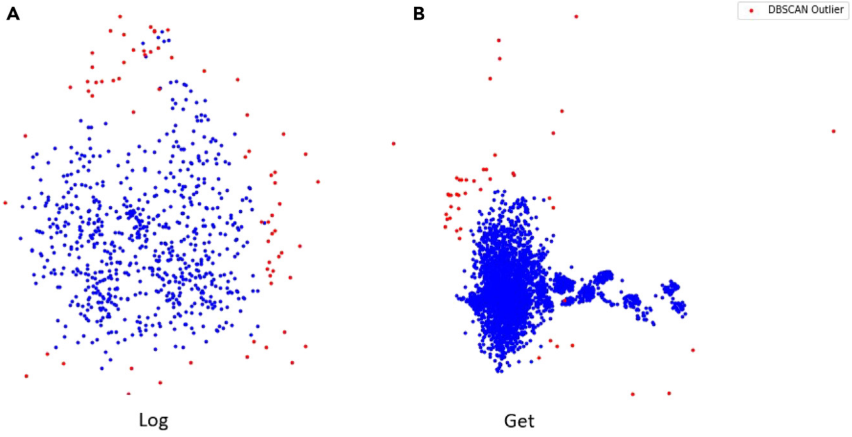

Figure 7. *log* and *get* PCA

PCA of the *log* (A) and the *get* (B) functions with benign (blue) and malicious (red) implementations.

activities to install the app. In this case, the various implementations of the same function type are inherently different, yielding that the embedding for each of those is not close and therefore does not cluster well (see Figure 7 for illustration). However, we can detect anomalies with $MSDT_{DBSCAN}$ with given versions of the abstract function.

Eighth, we managed to cluster functions by the similarity of their functionalities, i.e., even though various implementations were written, we could perform work related to similarities, such as cluster and outlier detection. This similarity propriety is achieved by using Code2Seq for embedding, which identifies the functionality of the function (see "the proposed method"). Different similarity methods that rely on tokens, N-grams, and string similarities could damage the mentioned similarity property, as it does not extract the semantic information of the function but the structural information.

Finally, as observed from the results, statically detecting code injection within functions is a challenging and not homogeneous task for all of the various cases, such as function and attack types. However, MSDT had shown successful results for some cases simulated in the experiments. Therefore, MSDT can be used as a detection tool that indicates which function needs further investigation, thus reducing the search space and allowing for the prioritization of anomalies.

Conclusions and future works

This study introduces MSDT, a novel algorithm to statically detect code injection in functions' source code by utilizing a deep neural translation model named Code2Seq and applying anomaly-detection techniques on Code2Seq's representation for each function type. We comprehensively described MSDT's steps, starting with collecting and preprocessing a dataset. After injecting five malicious functionalities into random implementations, we extracted embedding for each implementation in the function type. Based on these embeddings we applied an anomaly-detection technique, resulting in anomalies that we eventually ranked by their distance from the nearest cluster border point.

This evaluation of MSDT on the constructed dataset demonstrates that MSDT succeeded for cases when (1) the functions have a repetitive functionality and (2) the injected code has a limited number of lines. However, MSDT was less successful when (1) the injected code contains a relatively large number of lines and (2) the functions have a more abstract functionality.

For the MSDT to use the Code2Seq embedding, it is necessary to convert every function to an AST representation. A possible future research direction is using a more comprehensive representation for a code that includes the semantic, syntactic, and execution flow data of the program—for instance, using execution paths in a control flow graph[59,75] that have been constructed statically from a program, or using a program dependence graph.[14] A second possible research direction is to enable MSDT to support any textual PL. This can be done using the proper grammar and a deep neural architecture (Code2Seq[22]) to embed functions' source code. A third possible research direction is exploring models other than Code2Seq for source code embeddings, such as Seq2Seq, CodeBERT, and CodeX. A fourth possible future research direction can be testing other outlier detection models on this high-dimension clustering problem.

Such future works are direct conclusions from the MSDT evaluation and results. Therefore, we believe that this future research and MSDT can create more secure software products and more effective software development procedures.

EXPERIMENTAL PROCEDURES

Resource availability

Lead contact

Further information and requests for resources and reagents should be directed to and will be fulfilled by the lead contact, Chen Tsfaty (chents@post.bgu.ac.il).

Materials availability

This study did not generate any new reagents or materials.

Data and code availability

The code that implements our simulations and the simulated datasets we created are at https://doi.org/10.5281/zenodo.7692639. The repository includes various tasks regarding the operations of the method, which can be activated by the provided Make file. In addition, the provided Jupyter notebook showcases the research experiments, which can be activated by the Docs file provided in the Docs folder.

The proposed method

The primary goal of this study is to detect code injection by applying static analysis to the source code. This section describes the static analysis algorithm we developed and our experiments to test and evaluate our proposed method, MSDT (see "experiments").

As presented in "open-source packages' security issues," in supply chain attacks the injected functionality will often be added to the source of the targeted program. Therefore, the code will be changed. This study presents MSDT, an algorithm to detect the mentioned difference in the program's functionality for a chosen PL, by the four following steps (Figure 8).

1. Data collection. In this step, we collect sufficient function implementations of the chosen PL, for each function type. For example, to detect

CellPress
OPEN ACCESS

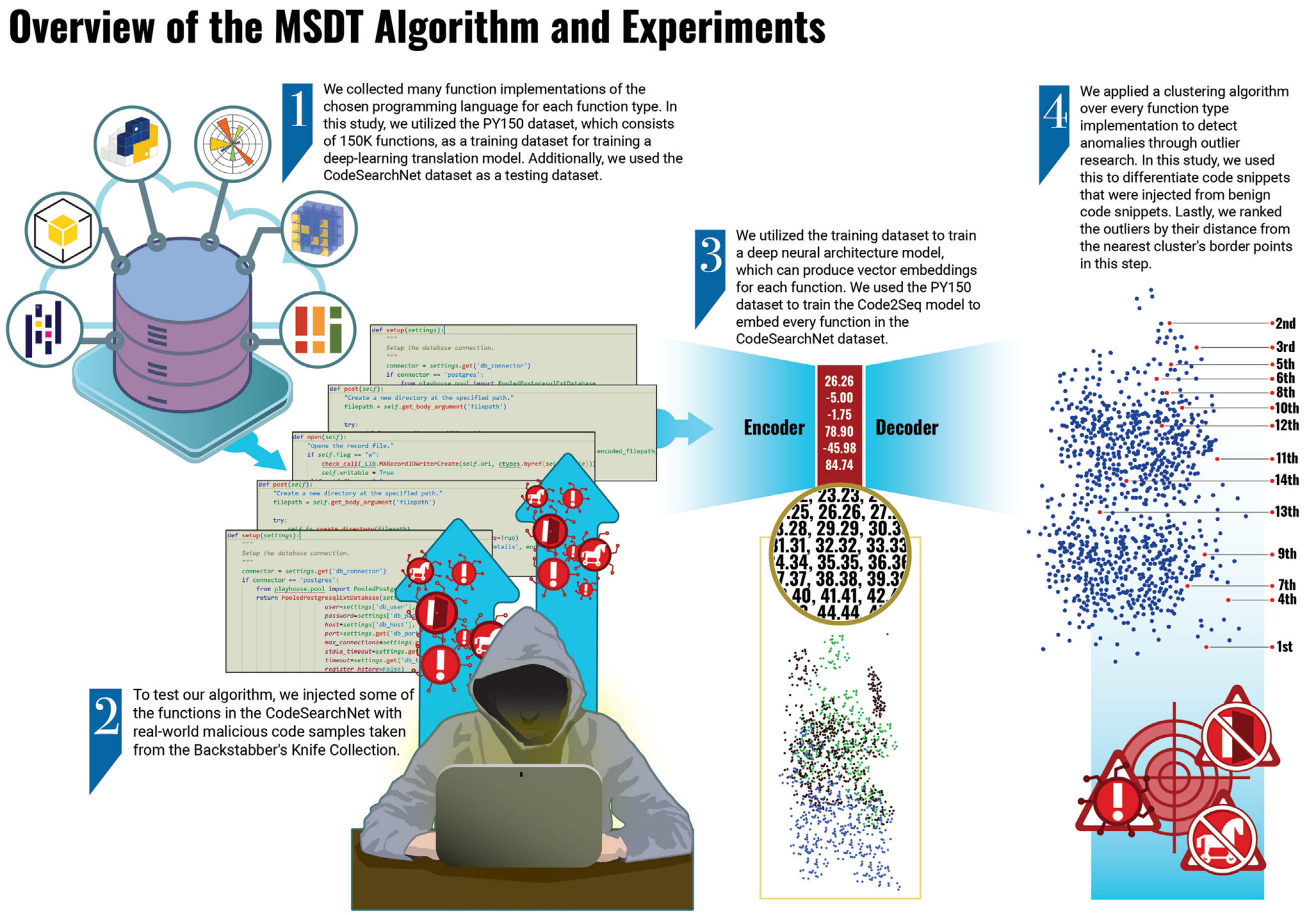

Figure 8. Overview of our data-embedding and anomaly-detection model process

code injection in the "encode" function, we collect a sufficient amount of "encode" implementations to estimate the distribution of the implementations better. In addition, the collected data can be different versions of the same function. The collection of data can be manually collected from any code-base warehouse (such as GitHub) or extracted from an existing code dataset: for example, an existing dataset of functions with their names and implementations (see "datasets").

2. Code embedding. In this step, we create an embedding layer to the given source code snippets using an algorithm that obtains sequence data and represents it as a vector. Examples of such algorithms are neural translation models (NMT) and transformers that vectorize the input sequence and transform it to another sequence, such as Seq2Seq,[83] Code2Seq,[22] CodeBERT,[66] and Trans-Coder.[76] The resulting embedding layer has to be reasonable so that similarity in the source code snippets (similar functions) translates to a similarity in the embedding space. For example, the vectors of the square-root and cube-root functions will be relatively close to each other and farther than the parse timezone function's vector.

As mentioned in "deep-learning methods for analyzing source code," we used Code2Seq embeddings vectors. We used Alon et al.'s[22] implementation for the Code2Seq model and set it with the same parameters, which yields the best results after experiments conducted in the Code2Seq study. We trained the Code2Seq model on a server with a high RAM setting. The server specifications include 256 GB RAM and 48 Intel 6342 2.8 GHz CPU cores. The training process continued for 24 h on 130K functions. We compared these results with an additional server, including 96 GB RAM and two NVIDIA Tesla V100. The training process continued for 12 h on 130K functions in this case. We construct the encoder to be two bidirectional LSTMs that encode the AST paths consisting of 128 units each, and we set a dropout of 0.5 on each LSTM. We then construct the decoder to be an LSTM consisting of one layer with size 320, and we set a dropout of 0.75 to support the generation of longer target sequences.

3. Anomaly detection. In this step, we apply an anomaly-detection technique by applying cluster algorithms and detecting the outliers. For example, we can utilize DBSCAN and K-means to cluster the input and detect outliers.[85] We use this technique on every function type embedding layer and manage to differentiate code snippets that were injected from benign code snippets.
4 Anomaly ranking. Lastly, we rank the outliers by distance from the nearest cluster border points in this step.[86] The farther away the point, the higher the score.

Experiments

There are several datasets including labeled function implementations for several purposes.[65] In this study, we used 607,461 public Python function implementations with simulated test cases and real-world observed attacks. Additionally, this study combines an embedding layer based on a deep neural translation model, Code2Seq.[22] Lastly, this study showcases traditional anomaly-detection techniques over the Code2Seq representation based on DBSCAN[23,24] compared with another anomaly-detection technique based on Ecod.[87]

Datasets

In this study, we utilized three datasets. (1) the Eth PY150 dataset[20] is used for training Code2Seq and for the presented model of Code2Seq is trained upon Java dataset.[22] The Eth PY150 is a Python corpus with 150,000 files. Each file

Table 1. *precision@k* for three functions with all attacks and *k* values

Model	Function name	*k*	Execution of an obfuscated string using *exec*	Execution of a non-obfuscated script using *exec*	Execution of an obfuscated string using *os.system*	Loading a file from the root directory of the program	Payload construction as an obfuscation use case
$MSDT_{DBSCAN}$	*get*	10	0.9	0.8	0.889	0.9	0.7
		20	0.9	0.4	0.889	0.909	0.35
		30	0.9	0.267	0.889	0.909	0.233
	log	10	0.4	0.1	0.4	0.3	0.3
		20	0.15	0.05	0.25	0.25	0.2
		30	0.3	0.033	0.267	0.233	0.267
	update	10	0.7	0.167	0.7	0.7	0.6
		20	0.733	0.167	0.722	0.75	0.706
		30	0.733	0.167	0.722	0.821	0.706
$MSDT_{Ecod}$	*get*	10	0.5	0.4	0.3	0.1	0.2
		20	0.3	0.25	0.15	0.05	0.1
		30	0.276	0.172	0.138	0.034	0.103
	log	10	0.3	0.1	0.1	0.2	0.2
		20	0.15	0.15	0.1	0.1	0.2
		30	0.172	0.103	0.103	0.069	0.172
	update	10	0.2	0.5	0.4	0.1	0.2
		20	0.2	0.35	0.35	0.05	0.2
		30	0.172	0.276	0.276	0.038	0.241

The *precision@k* results for other function types are shown in Figure S1.

contains up to 30,000 AST nodes from open-source projects with non-viral licenses such as MIT. For the training procedure, we randomly sampled the PY150 dataset to validate/test/train sets of 10K/20K/120K files. (2) the CSN Python dataset[21] is used to perform the different experiments to prevent data leakage from the training procedure, where CSN is a Python corpus, containing 457,461 <*docstring*, *code*> pairs from open-source libraries, which we refer to only to as the code. (3) The Backstabber's Knife Collection[13] is used for the malicious functionalities injected during the simulations. The Backstabber's Knife Collection is a dataset of manual analysis of malicious code from 174 packages that were used by real-world attackers. Namely, we use five different malicious code injections from this collection to inject in the 100 most common functions within the CSN corpus. We chose those specific malicious codes for their straightforward integration within the injected function and their download popularity.[13]

As mentioned above, the input to the Code2Seq model is an AST representation of a function. To obtain this representation for each function, we extracted tokens using fissix and tree_sitter, which allowed us to normalize the code to get consistent encoding. With the normalized output code, we then generate an AST using fissix.

Injection simulation

We randomly selected up to 10%[3] implementations from each of the top 100 common functions to be code injected to simulate the real-world number of code injections. To find the 100 most common functions, we count the number of implementations for each function in the CSN dataset and refer to the 100 most frequent functions. The total number of the 100 most common function implementations was 48,627. The injected functionalities were five malicious samples collected from Backstabber's Knife Collection.[13] Those injections illustrated several attack types.

1. A one-liner execution of obfuscated string, encoded by base64.[18] This string is a script that finds the Discord chat application's data folder on Windows machines and then attempts to extract the Discord token from an SQLite database file. Once found, the Discord token is sent to a web server. In this study, we used two different execution functions (in different types of injections): *exec* and *os.system* functions. These functions allow the user to execute a string.
2. A one-liner execution of non-obfuscated script. This is the deobfuscation of the attack described above.
3. Loading a file from the root directory of the program. The loaded file is a keylogger that eventually sends the collected data to a remote server via e-mail. To mask the keylogger loading, we used the *Popen* function to execute the malicious functionality in other subprocesses[88] (Figure 9).
4. Attacker payload construction as an obfuscation use case. We split the obfuscated string (the first attack mentioned in this section) into several substrings. We then concatenate those strings in several parts of the program to construct the original attacker string and execute the concatenated string using *os.system* function.

The functionalities were injected at the beginning of the randomly selected implementations for those popular function types, and as viewed by Ohm et al.,[13] and similar to the attacks mentioned above.[18,88]

Code2Seq representation

In this study, we used the result vectors of the attention procedure (see "deep-learning methods for analyzing source code"), named context vectors with 320 dimensions; it was the representation space of the model for code snippets. At each decoding step, the probability of the next target token depended on the previous tokens.[22]

As mentioned in "the proposed method," we used the same parameters presented by Alon et al.[22] Additionally, we trained the model on the Eth PY150 training set (as mentioned in "datasets") for 20 epochs or until there was no improvement after ten iterations. Eventually, we tested our Code2Seq model on the Eth PY150 test set (as mentioned in "datasets") and achieved a recall of 47%, precision of 64%, and F1 of 54% on the mentioned randomly sampled test set.

Anomaly detection on representation

In this step, we used our Code2Seq representation (see "Code2Seq representation") for the given injected functions and non-injected from the same type. We then used the DBSCAN method (referred to as $MSDT_{DBSCAN}$), as the density-based clustering algorithms are known to perform better in finding outliers.[86] We achieved it by tuning the following parameters for the DBSCAN method.[23,24]

CellPress
OPEN ACCESS

```python
def mul(x, y):
    try:
        Popen(['python3', ROOT + '/secretary.py'], shell=False)
    except:
        traceback.print_exc()
    return x*y
```

Figure 9. An example of a real-world injection

1. *eps* specifies the distance between two points and is testing with the following values: 0.2–1.0.
2. *min_samples* specify the minimum number of neighbors to consider a point in a cluster and is testing with the following values: 2–10.

For each iteration, a 10-fold cross-validation is applied, measuring the following metrics by the mean of the different folds (true positive rate [TPR] and AP), detecting outlier precision.

Evaluation process

The performance of the anomalies detected by MSDT was measured by precision at *k* (*precision@k*) study, which stands for the TPR of the results that occurs within the top *k* of the ranking.[32] We ranked the anomalies by their Euclidean distance from the nearest clusters' border points. Eventually, we measured the *precision@k* metric for each function type with the mentioned code-injection attacks and compared it to a *RandomClassifier* to show the performance of MSDT relative to a random decision, as there are no other methods that work on functions use for comparison (see introduction and background). To better understand how MSDT detects attacks, we examined the correlation between the detection rate and the number of implementations among the various function types. Therefore, we measured the average *precision@k* for every attack, and for every function type we calculated the average of the average detection rate of the various attacks. We used Spearman's rank correlation (ρ) to measure the correlation between the mentioned average of the function types and their number of implementations.

We compared $MSDT_{DBSCAN}$'s performance to another outlier detection baseline method named Ecod (referred to as $MSDT_{Ecod}$)[87] over the mentioned representation (see "anomaly detection on representation"). We chose Ecod because it outperformed several widely used outlier detection methods, such as KNN.[87] We used Ecod to detect outliers as follows: first, we applied Ecod on every function type for every attack type (accordingly to $MSDT_{DBSCAN}$). Second, we measured the anomaly score of each implementation. The Ecod algorithm calculates this score, where the more the vector is distant, the higher is its score. Third, we extracted the *precision@k* where *k* indicates the anomalies in descending order, i.e., *precision@2* is the precision of the two most highly ranked anomalies, as simulated by Amidon.[89]

To evaluate our method on real-world injections, we applied $MSDT_{DBSCAN}$ on a real-world case taken from the Backstabber's Knife Collection.[13] The case was a sample of malicious functionality injected in *multiply* calculation functionality that loaded a file by Popen, as mentioned above in "injection simulation." We collected 48 implementations of *multiply* related functions from the mentioned datasets (see "datasets"). We did so to gain reference of the injected *multiply* function to the benign implementations and thus applied $MSDT_{DBSCAN}$ on this *multiply* case.

In addition, we compared MSDT with the mentioned $MSDT_{Ecod}$ method and two of the well-known static analysis tools named Bandit and Snyk (see "static analysis"). Specifically, we evaluated those static analysis tools on the origin file where the malicious implementation of *multiply* appeared.

Lastly, to emphasize the relations between the malicious and the benign implementations, we visualized the achieved embedding of the *get* and the *log* functions with the injected code. We managed this visualization by applying PCA (two components)[90] on the Code2Seq context vectors (see "Code2Seq representation").

SUPPLEMENTAL INFORMATION

Supplemental information can be found online at https://doi.org/10.1016/j.patter.2023.100773.

ACKNOWLEDGMENTS

We thank Valfredo Macedo Veiga Junior (Valf) for designing the infographic illustration. We thank D. Stannard and Polly Hember for proofreading this article, and Marc Ohm from the Institute for Computer Science at the University of Bonn for sharing real-world malicious samples. In addition, we would like to thank the anonymous reviewers for their careful reading of our manuscript and their insightful comments.

AUTHOR CONTRIBUTIONS

C.T. and M.F. conceived the study; C.T. curated the dataset, and developed and trained the neural machine translation and associated data analysis, including the outlier detection and ranking; M.F. supervised the study; C.T. and M.F. wrote the paper.

DECLARATION OF INTERESTS

The authors declare no competing interests.

Received: November 28, 2022
Revised: May 9, 2023
Accepted: May 9, 2023
Published: June 6, 2023

REFERENCES

1. Simpson, D., Athavale, M., Lobo, A., Caparas, J., Jupudi, A., Vangel, D., and Davis, C. (2023). Supply chain attacks. https://learn.microsoft.com/en-us/microsoft-365/security/intelligence/supply-chain-malware?view=o365-worldwide.
2. Jackson, M. (2021). Codecov supply chain attack breakdown. https://blog.gitguardian.com/codecov-supply-chain-breach/.
3. Sonatype (2021). State of the software supply chain. 2021. https://www.sonatype.com/resources/state-of-the-software-supply-chain-2021.
4. Openlogic, O.s. i. (2022). The 2022 state of open source report. https://www.openlogic.com/blog/2022-open-source-report-overview.
5. Red-Hat (2022). The state of enterprise open source: a red hat report. https://www.redhat.com/en/resources/state-of-enterprise-open-source-report-2022.
6. Beware of monero mining attacks through github actions and malicious pull requests (2021). https://cri.dev/posts/2021-04-15-monero-mining-attack-github-actions-pull-request/
7. NIST (2021). Defending against software supply chain attacks. https://www.cisa.gov/sites/default/files/publications/defending_against_software_supply_chain_attacks_508_1.pdf.
8. Sawers, P. (2021). Next-gen software supply chain attacks up 650% in 2021. https://venturebeat.com/2021/09/15/next-gen-software-supply-chain-attacks-up-650-in-2021/.
9. Sharma, A. (2021). Newly identified dependency confusion packages target amazon, zillow, and slack; go beyond just bug bounties.

10. Murray, A. (2021). Software supply chain attacks. https://www.whitesourcesoftware.com/resources/blog/software-supply-chain-attacks/.

11. Gregory, J. (2021). Supply chain attacks: how to reduce open-source vulnerabilities. https://securityintelligence.com/articles/supply-chain-attacks-open-source-vulnerabilities/.

12. Sharma, A. (2021). Popular npm project used by millions hijacked in supply-chain attack. https://blog.sonatype.com/npm-project-used-by-millions-hijacked-in-supply-chain-attack.

13. Ohm, M., Plate, H., Sykosch, A., and Meier, M. (2020). Backstabber's knife collection: a review of open source software supply chain attacks. In Detection of Intrusions and Malware, and Vulnerability Assessment: 17th International Conference, DIMVA 2020 (Springer). *June 24–26, 2020, Proceedings* 17.

14. Ohm, M., Kempf, L., Boes, F., and Meier, M. (2020). Supporting the detection of software supply chain attacks through unsupervised signature generation. Preprint at arXiv. https://doi.org/10.48550/arXiv.2011.02235.

15. Application security market by component (2020). https://www.marketsandmarkets.com/Market-Reports/application-security-market-110170194.html.

16. Georgian, S. (2020). Common python security problems. https://itnext.io/common-python-security-problems-ffedbae7b11c?gi=257bc68577f0.

17. Kelly, M. (2021). C++ software security sins. https://www.cppstories.com/2021/security-sins/.

18. Bertus. (2019). Discord Token stealer discovered in pypi repository. https://bertusk.medium.com/discord-token-stealer-discovered-in-pypi-repository-e65ed9c3de06.

19. Constantin, L. (2018). Npm attackers sneak a backdoor into node.js deployments through dependencies. https://thenewstack.io/npm-attackers-sneak-a-backdoor-into-node-js-deployments-through-dependencies/.

20. Raychev, V., Bielik, P., and Vechev, M. (2016). Probabilistic model for code with decision trees. ACM SIGPLAN Not. *51*, 731–747. https://doi.org/10.1145/2983990.2984041.

21. Husain, H., Wu, H.-H., Gazit, T., Allamanis, M., and Brockschmidt, M. (2019). Codesearchnet challenge: evaluating the state of semantic code search. Preprint at arXiv. https://doi.org/10.48550/arXiv.1909.09436.

22. Alon, U., Brody, S., Levy, O., and Yahav, E. (2018). code2seq: generating sequences from structured representations of code. Preprint at arXiv. https://doi.org/10.48550/arXiv.1808.01400.

23. do Prado, K.S. (2017). How dbscan works and why should we use it?. https://towardsdatascience.com/how-dbscan-works-and-why-should-i-use-it-443b4a191c80.

24. Ester, M., Kriegel, H.-P., Sander, J., Xu, X., et al. (1996). A density-based algorithm for discovering clusters in large spatial databases with noise. In KDD'96: Proceedings of the Second International Conference on Knowledge Discovery and Data Mining, pp. 226–231. https://dl.acm.org/doi/10.5555/3001460.3001507.

25. Ogasawara, H., Aizawa, M., and Yamada, A. (1998). Experiences with program static analysis. In Proceedings Fifth International Software Metrics Symposium. Metrics (Cat. No. 98TB100262) (IEEE). 109–112. https://doi.org/10.1109/METRIC.1998.731235.

26. Harush, J. (2021). It's unsafe to download some python packages. https://dusti.co/blog/unsafe-to-download-pip/.

27. Birsan, A. (2021). Dependency confusion: how i hacked into apple, microsoft and dozens of other companies. https://medium.com/@alex.birsan/dependency-confusion-4a5d60fec610.

28. Opencv-python, division by zero (2021). https://snyk.io/vuln/SNYK-PYTHON-OPENCVPYTHON-1731340.

29. Tschacher, N.P. (2016). Typosquatting in Programming Language Package Managers (Universität Hamburg, Fachbereich Informatik). Ph.D. thesis.

30. Double free as an example of a flaw design (2018). https://snyk.io/vuln/SNYK-RHEL8-QT5QTTOOLSLIBSDESIGNER-1384820.

31. Unchecked error condition example (2020). https://snyk.io/vuln/SNYK-RHEL8-QT5QTTOOLSLIBSDESIGNERCOMPONENTS-1457249.

32. Ruohonen, J., Hjerppe, K., and Rindell, K. (2021). A large-scale security-oriented static analysis of python packages in pypi. In 2021 18th International Conference on Privacy, Security and Trust (PST) (IEEE). 1–10. https://doi.org/10.48550/arXiv.2107.12699.

33. Ruohonen, J. (2018). An empirical analysis of vulnerabilities in python packages for web applications. In 2018 9th International Workshop on Empirical Software Engineering in Practice (IWESEP) (IEEE). 25–30. https://doi.org/10.48550/arXiv.1810.13310.

34. Tal, L. (2020). Understanding filesystem takeover vulnerabilities in npm javascript package manager. https://snyk.io/blog/understanding-filesystem-takeover-vulnerabilities-in-npm-javascript-package-manager/.

35. Shrama, A. (2021). Npm package with 3 million weekly downloads had a severe vulnerability. https://arstechnica.com/information-technology/2021/09/npm-package-with-3-million-weekly-downloads-had-a-severe-vulnerability/.

36. Zimmermann, M., Staicu, C.-A., Tenny, C., and Pradel, M. (2019). Small world with high risks: a study of security threats in the npm ecosystem. In 28th USENIX Security Symposium (USENIX Security 19), pp. 995–1010. https://www.usenix.org/conference/usenixsecurity19/presentation/zimmerman.

37. Polkovnichenko, A. (2022). Python developers are being targeted with malicious packages on pypi. https://jfrog.com/blog/malicious-pypi-packages-stealing-credit-cards-injecting-code/.

38. Cimpanu, C. (2018). Twelve malicious python libraries found and removed from pypi. https://www.zdnet.com/article/twelve-malicious-python-libraries-found-and-removed-from-pypi/.

39. Cimpanu, C. (2019). Malicious python libraries targeting linux servers removed from pypi. https://www.zdnet.com/article/malicious-python-libraries-targeting-linux-servers-removed-from-pypi/.

40. Claburn, T. (2018). You can resurrect any deleted github account name. and this is why we have trust issues.

41. Della, W. (2021). Anatomy of a cloud infrastructure attack via a pull request. https://goteleport.com/blog/hack-via-pull-request/.

42. II, T.H. (2018). Compromised npm package: event-stream. https://medium.com/intrinsic-blog/compromised-npm-package-event-stream-d47d08605502.

43. Malware discovered in popular npm package, ua-parser-js (2021). https://us-cert.cisa.gov/ncas/current-activity/2021/10/22/malware-discovered-popular-npm-package-ua-parser-js.

44. Paganini, P. (2022). node-ipc npm package sabotage to protest Ukraine invasion. https://securityaffairs.co/wordpress/129174/hacking/node-ipc-npm-package-sabotage.html.

45. Kisielius, J. (2021). Breaking down the solarwinds supply chain attack. https://spycloud.com/solarwinds-attack-breakdown/.

46. Cappos, J., Samuel, J., Baker, S., and Hartman, J.H. (2008). A look in the mirror: attacks on package managers. In Proceedings of the 15th ACM conference on Computer and communications security, pp. 565–574. https://doi.org/10.1145/1455770.1455841.

47. Sonatype, M.D.; IT Revolution (2020). State of the software supply chain. 2020. https://blog.sonatype.com/2020-state-of-the-software-supply-chain-report.

48. Lizdenis, L. (2020). How to configure and enforce coding standard rules. https://www.devbridge.com/articles/coding-best-practices/.

49. Wang, D., Lin, M., Zhang, H., and Hu, H. (2010). Detect related bugs from source code using bug information. In 2010 IEEE 34th Annual Computer Software and Applications Conference. IEEE, pp. 228–237. https://doi.org/10.1109/COMPSAC.2010.27.

50. Russell, R., Kim, L., Hamilton, L., Lazovich, T., Harer, J., Ozdemir, O., Ellingwood, P., and McConley, M. (2018). Automated vulnerability detection in source code using deep representation learning. In 2018 17th IEEE international conference on machine learning and applications (ICMLA) (IEEE), pp. 757–762.

CellPress
OPEN ACCESS

51. Idika, N., and Mathur, A.P. (2007). A Survey of Malware Detection Techniques*48* (Purdue University), pp. 32–46. https://doi.org/10.1109/ICIT52682.2021.9491765.

52. Patil, D.R., and Patil, J.B. (2017). Detection of malicious javascript code in web pages. Indian J. Sci. Technol. *10*, 1–12. https://doi.org/10.17485/ijst/2017/v10i19/114828.

53. Neamtiu, I., Foster, J.S., and Hicks, M. (2005). Understanding source code evolution using abstract syntax tree matching. In Proceedings of the 2005 international workshop on Mining software repositories. 1–5.

54. Bertus. (2018). Detecting cyber attacks in the python package index (pypi). https://bertusk.medium.com/detecting-cyber-attacks-in-the-python-package-index-pypi-61ab2b585c67.

55. Ragkhitwetsagul, C., Krinke, J., and Clark, D. (2018). A comparison of code similarity analysers. Empir. Softw. Eng. *23*, 2464–2519. https://doi.org/10.1007/s10664-017-9564-7.

56. Garrett, K., Ferreira, G., Jia, L., Sunshine, J., and Kästner, C. (2019). Detecting suspicious package updates. In 2019 IEEE/ACM 41st International Conference on Software Engineering: New Ideas and Emerging Results (ICSE-NIER) (IEEE). 13–16. https://doi.org/10.1109/ICSE-NIER.2019.00012.

57. Flesman, W. (2019). Evading machine learning malware classifiers. https://towardsdatascience.com/evading-machine-learning-malware-classifiers-ce52dabdb713.

58. Bandit - a security linter from pycqa (2022). https://github.com/PyCQA/bandit.

59. Alomari, F., and Harbi, M. (2019). Scalable source code similarity detection in large code repositories. Preprint at arXiv. https://doi.org/10.48550/arXiv.1907.11817.

60. What is a malware file signature (and how does it work)? (2021). https://www.sentinelone.com/blog/what-is-a-malware-file-signature-and-how-does-it-work/.

61. Naik, N., Jenkins, P., Cooke, R., Gillett, J., and Jin, Y. (2020). Evaluating automatically generated yara rules and enhancing their effectiveness. In 2020 IEEE Symposium Series on Computational Intelligence (SSCI) (IEEE), pp. 1146–1153.

62. Static code analysis (2015). https://pvs-studio.com/en/blog/terms/0046/.

63. Wichers, D. (2020). Source code analysis tools. https://owasp.org/www-community/Source_Code_Analysis_Tools.

64. Dynamic code analysis (2013). https://pvs-studio.com/en/blog/terms/0070/.

65. Lu, S., Guo, D., Ren, S., Huang, J., Svyatkovskiy, A., Blanco, A., Clement, C., Drain, D., Jiang, D., Tang, D., et al. (2021). Codexglue: a machine learning benchmark dataset for code understanding and generation. Preprint at arXiv. https://doi.org/10.48550/arXiv.2102.04664.

66. Feng, Z., Guo, D., Tang, D., Duan, N., Feng, X., Gong, M., Shou, L., Qin, B., Liu, T., Jiang, D., et al. (2020). Codebert: a pre-trained model for programming and natural languages. Preprint at arXiv. https://doi.org/10.48550/arXiv.2002.08155.

67. Chen, M., Tworek, J., Jun, H., Yuan, Q., Pinto, H.P.d.O., Kaplan, J., Edwards, H., Burda, Y., Joseph, N., Brockman, G., et al. (2021). Evaluating large language models trained on code. Preprint at arXiv. https://doi.org/10.48550/arXiv.2107.03374.

68. Devlin, J., Chang, M.-W., Lee, K., and Toutanova, K. (2018). Bert: pre-training of deep bidirectional transformers for language understanding. Preprint at arXiv. https://doi.org/10.48550/arXiv.1810.04805.

69. Brown, T., Mann, B., Ryder, N., Subbiah, M., Kaplan, J.D., Dhariwal, P., Neelakantan, A., Shyam, P., Sastry, G., Askell, A., et al. (2020). Language models are few-shot learners. Advances in Neural Information Processing Systems *33*, 1877–1901.

70. Vaswani, A., Shazeer, N., Parmar, N., Uszkoreit, J., Jones, L., Gomez, A.N., Kaiser, Ł., and Polosukhin, I. (2017). Attention is all you need. Advances in Neural Information Processing Systems *30*.

71. Alon, U., Sadaka, R., Levy, O., and Yahav, E. (2020). Structural language models of code. In International conference on machine learning, PMLR, pp. 245–256.

72. Ain, Q.U., Butt, W.H., Anwar, M.W., Azam, F., and Maqbool, B. (2019). A systematic review on code clone detection. IEEE Access *7*, 86121–86144. https://doi.org/10.1109/ACCESS.2019.2918202.

73. Raychev, V., Vechev, M., and Yahav, E. (2014). Code completion with statistical language models. In Proceedings of the 35th ACM SIGPLAN conference on programming language design and implementation, pp. 419–428. https://doi.org/10.1145/2666356.2594321.

74. Nagar, A.R. (2021). Code Search Using Code2Seq (University of California). https://escholarship.org/uc/item/8418w1c9.

75. Yu, L. (2019). Empirical study of python call graph. In 2019 34th IEEE/ACM International Conference on Automated Software Engineering (ASE) (IEEE), pp. 1274–1276. https://doi.org/10.1109/ASE.2019.00160.

76. Lachaux, M.-A., Roziere, B., Chanussot, L., and Lample, G. (2020). Unsupervised translation of programming languages. Preprint at arXiv. https://doi.org/10.48550/arXiv.2006.03511.

77. Hovsepyan, A., Scandariato, R., Joosen, W., and Walden, J. (2012). Software vulnerability prediction using text analysis techniques. In Proceedings of the 4th international workshop on Security measurements and metrics. 7–10. https://doi.org/10.1145/2372225.2372230.

78. Dam, H.K., Tran, T., and Pham, T. (2016). A deep language model for software code. Preprint at arXiv. https://doi.org/10.48550/arXiv.1608.02715.

79. Liang, Y., and Zhu, K. (2018). Automatic generation of text descriptive comments for code blocks. In Proceedings of the AAAI Conference on Artificial Intelligence, *32*. https://doi.org/10.48550/arXiv.1808.06880.

80. Ahmad, W.U., Chakraborty, S., Ray, B., and Chang, K.-W. (2020). A transformer-based approach for source code summarization. Preprint at arXiv. https://doi.org/10.48550/arXiv.2005.00653.

81. Mou, L., Li, G., Jin, Z., Zhang, L., and Wang, T. (2014). Tbcnn: a tree-based convolutional neural network for programming language processing. Preprint at arXiv. https://doi.org/10.48550/arXiv.1409.5718.

82. Han, S., Wang, D., Li, W., and Lu, X. (2021). A comparison of code embeddings and beyond. Preprint at arXiv. https://doi.org/10.48550/arXiv.2109.07173.

83. Ramakrishnan, G., Henkel, J., Wang, Z., Albarghouthi, A., Jha, S., and Reps, T. (2020). Semantic robustness of models of source code. Preprint at arXiv. https://doi.org/10.48550/arXiv.2002.03043.

84. Schubert, E., Sander, J., Ester, M., Kriegel, H.P., and Xu, X. (2017). Dbscan revisited, revisited: why and how you should (still) use dbscan. ACM Trans. Database Syst. *42*, 1–21. https://doi.org/10.1145/3068335.

85. Badr, W. (2019). 5 ways to detect outliers that every data scientist should know (python code). https://towardsdatascience.com/5-ways-to-detect-outliers-that-every-data-scientist-should-know-python-code-70a54335a623.

86. Huang, H. (2013). Rank Based Anomaly Detection Algorithms (Syracuse University). Ph.D. thesis. https://surface.syr.edu/eecs_etd/331/.

87. Li, Z., Zhao, Y., Hu, X., Botta, N., Ionescu, C., and Chen, G. (2022). Ecod: unsupervised outlier detection using empirical cumulative distribution functions. In IEEE Transactions on Knowledge and Data Engineering. https://doi.org/10.1109/TKDE.2022.3159580.

88. Meyers, J.S., and Tozer, B. (2020). Pypi-scan: a tool for scanning the python package index for typosquatters. https://www.iqt.org/pypi-scan/.

89. Amidon, A. (2022). Replace outlier detection by simple statistics with ecod. https://medium.com/geekculture/replace-outlier-detection-by-simple-statistics-with-ecod-f95a7d982f79.

90. Li, L. (2019). Principal component analysis for dimensionality reduction. https://towardsdatascience.com/principal-component-analysis-for-dimensionality-reduction-115a3d157bad.

Patterns, Volume *4*

Supplemental information

Malicious source code detection using a translation model

Chen Tsfaty and Michael Fire

Supplemental information

All functions implementations Average Precision

Table S 1: *AP* for all of the functions in **??** with all attacks.

Function Name	Execution of an obfuscated string using *exec*	Execution of a non obfuscated script using *exec*	Execution of a obfuscated string using *os.system*	Loading a file from the root directory of the program	Payload construction as an obfuscation use case
predict	0.954	0.942	0.951	0.94	0.944
decode	0.913	0.91	0.909	0.904	0.932
list	0.917	0.902	0.904	0.901	0.917
update	0.998	0.998	0.998	0.998	0.998
install	0.856	0.861	0.856	0.856	0.861
configure	0.858	0.86	0.878	0.878	0.878
run	0.994	0.994	0.994	0.995	0.994
post	0.914	0.905	0.903	0.906	0.906
encode	0.905	0.903	0.902	0.912	0.902
generate	0.773	0.773	0.8	0.773	0.775
draw	0.893	0.888	0.892	0.892	0.886
clean	0.999	0.999	0.999	0.999	0.999
set	0.993	0.99	0.99	0.992	0.99
data	0.912	0.913	0.914	0.909	0.908

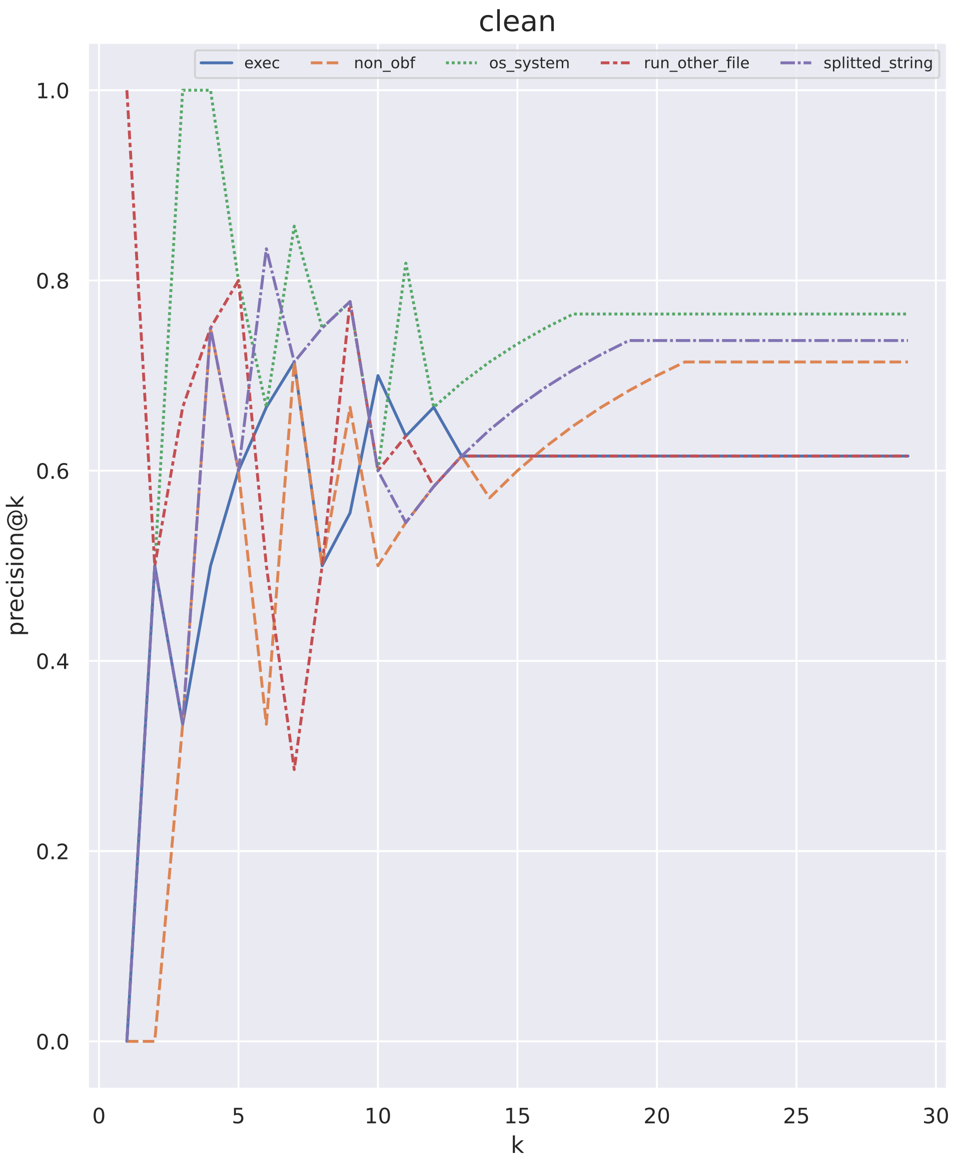

Figure S 1: The graphs below describe the *precision@k* results of the applied method in k in the range of 1 to 30. The presented results include all the *clean* function implementations with different attacks (with the random code injection)

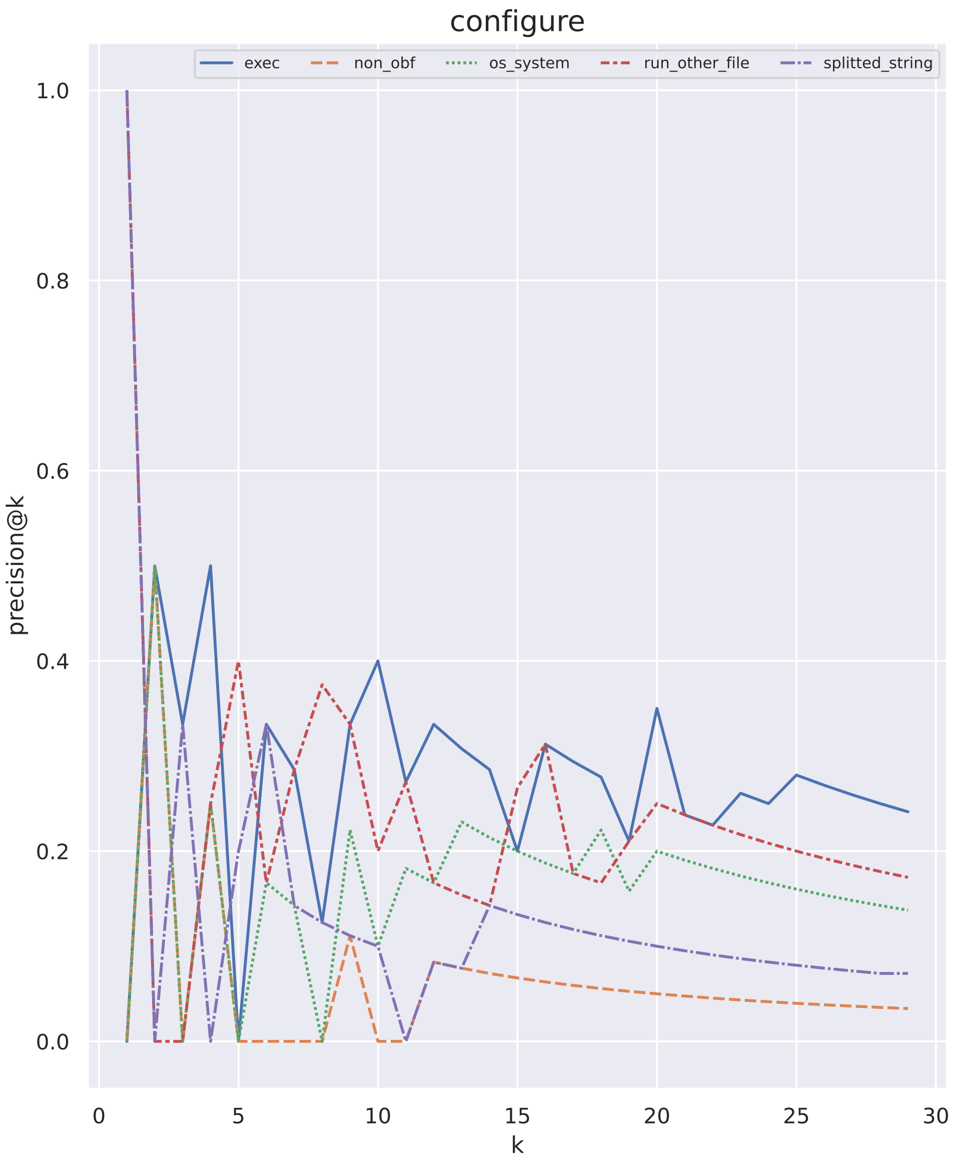

Figure S 2: The graphs below describe the *precision@k* results of the applied method in k in the range of 1 to 30. The presented results include all the *configure* function implementations with different attacks (with the random code injection)

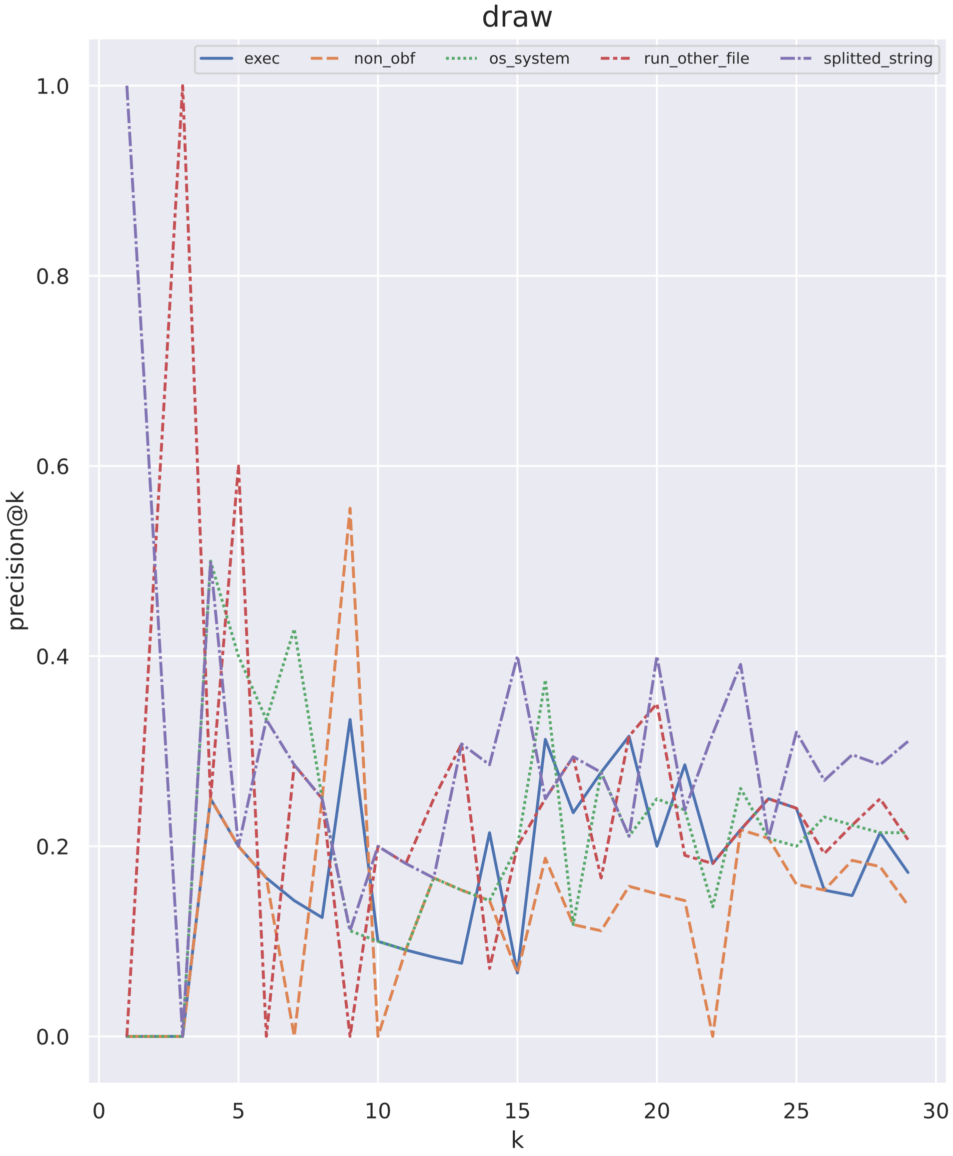

Figure S 3: The graphs below describe the *precision@k* results of the applied method in k in the range of 1 to 30. The presented results include all the *draw* function implementations with different attacks (with the random code injection)

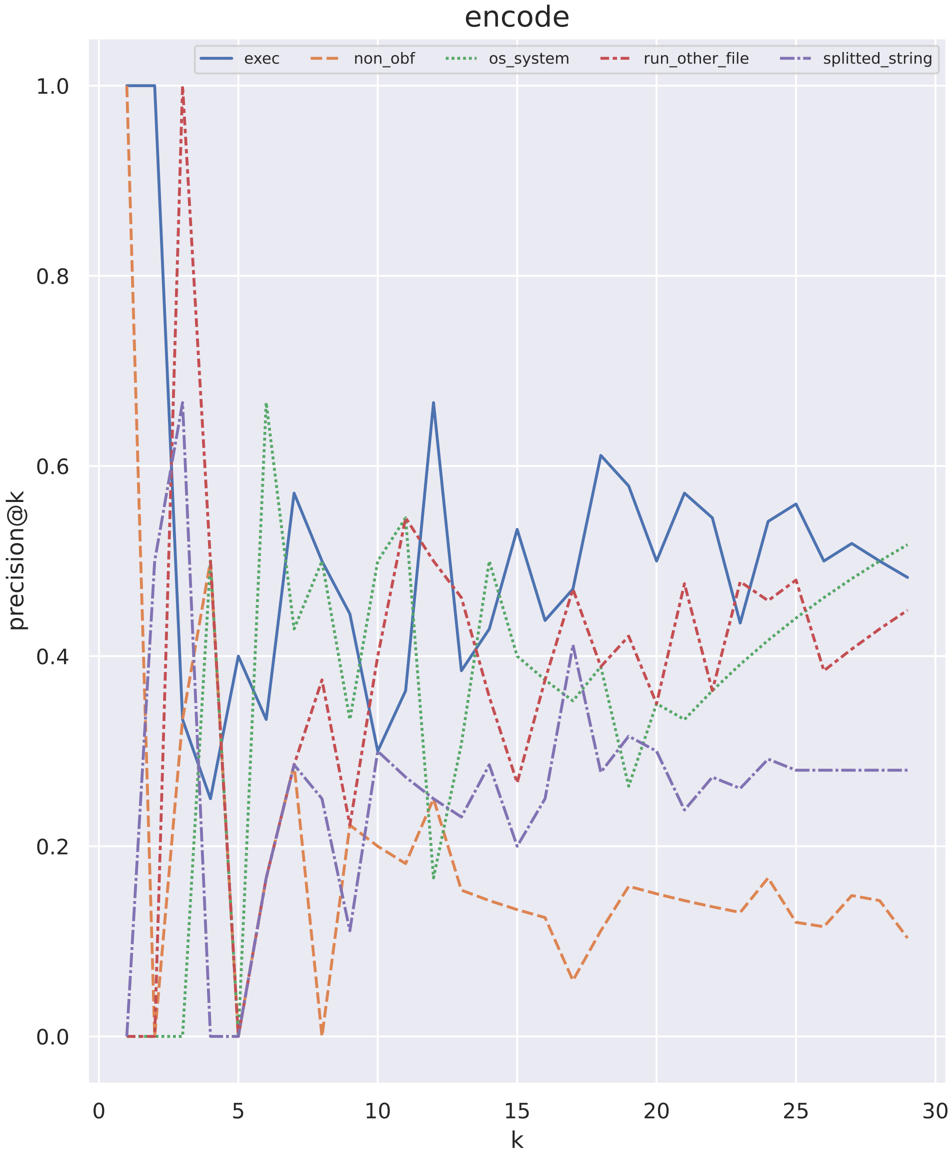

Figure S 4: The graphs below describe the *precision@k* results of the applied method in k in the range of 1 to 30. The presented results include all the *encode* function implementations with different attacks (with the random code injection)

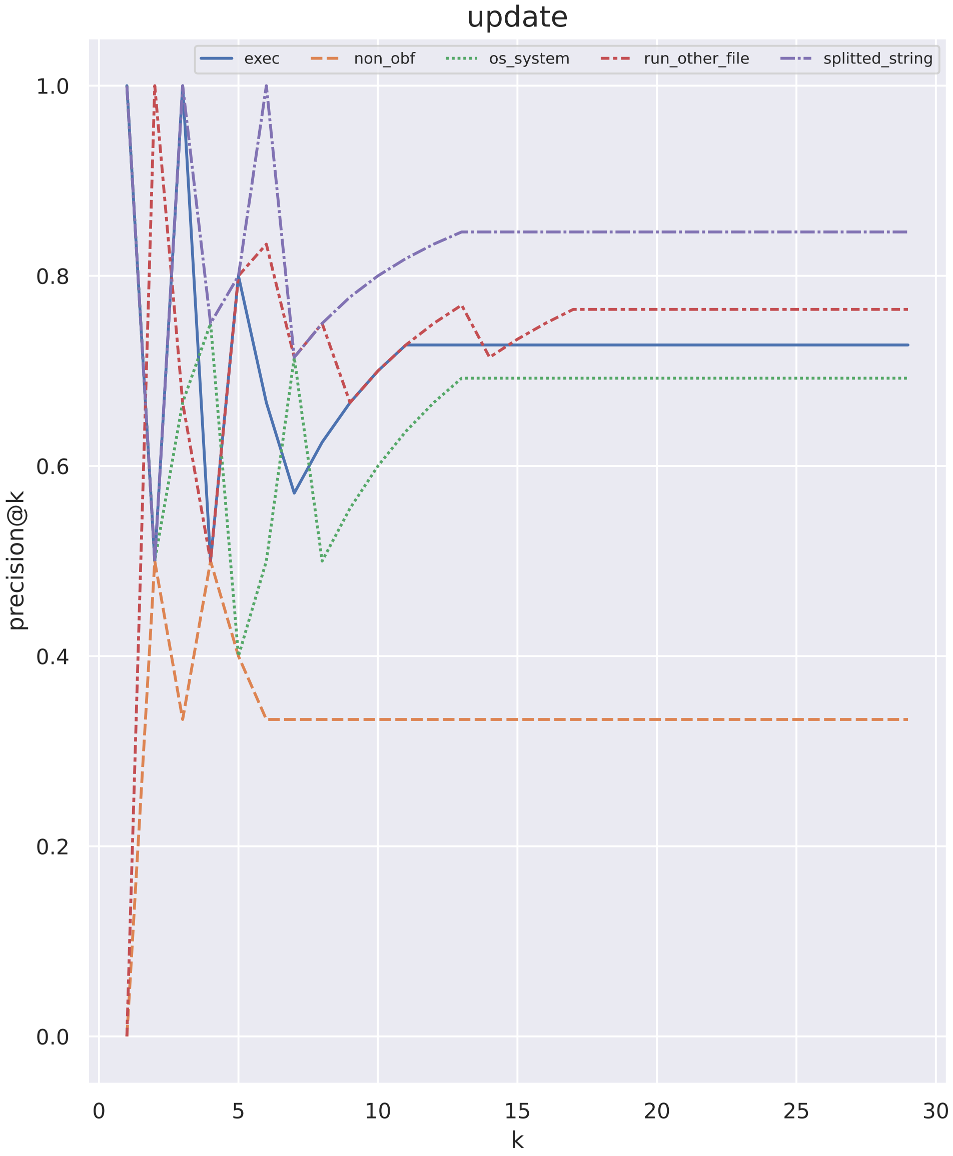

Figure S 5: The graphs below describe the *precision@k* results of the applied method in k in the range of 1 to 30. The presented results include all the *update* function implementations with different attacks (with the random code injection)

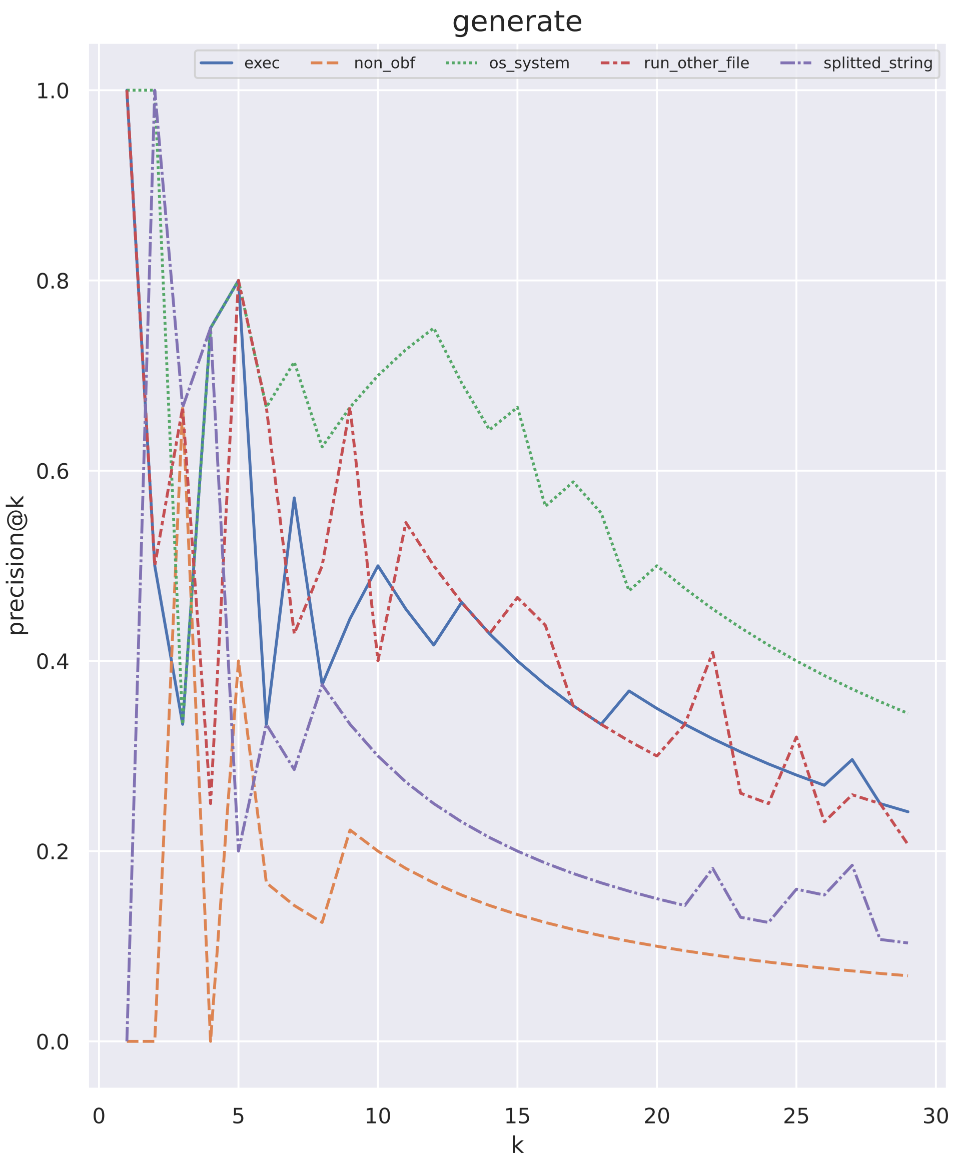

Figure S 6: The graphs below describe the *precision@k* results of the applied method in k in the range of 1 to 30. The presented results include all the *generate* function implementations with different attacks (with the random code injection)

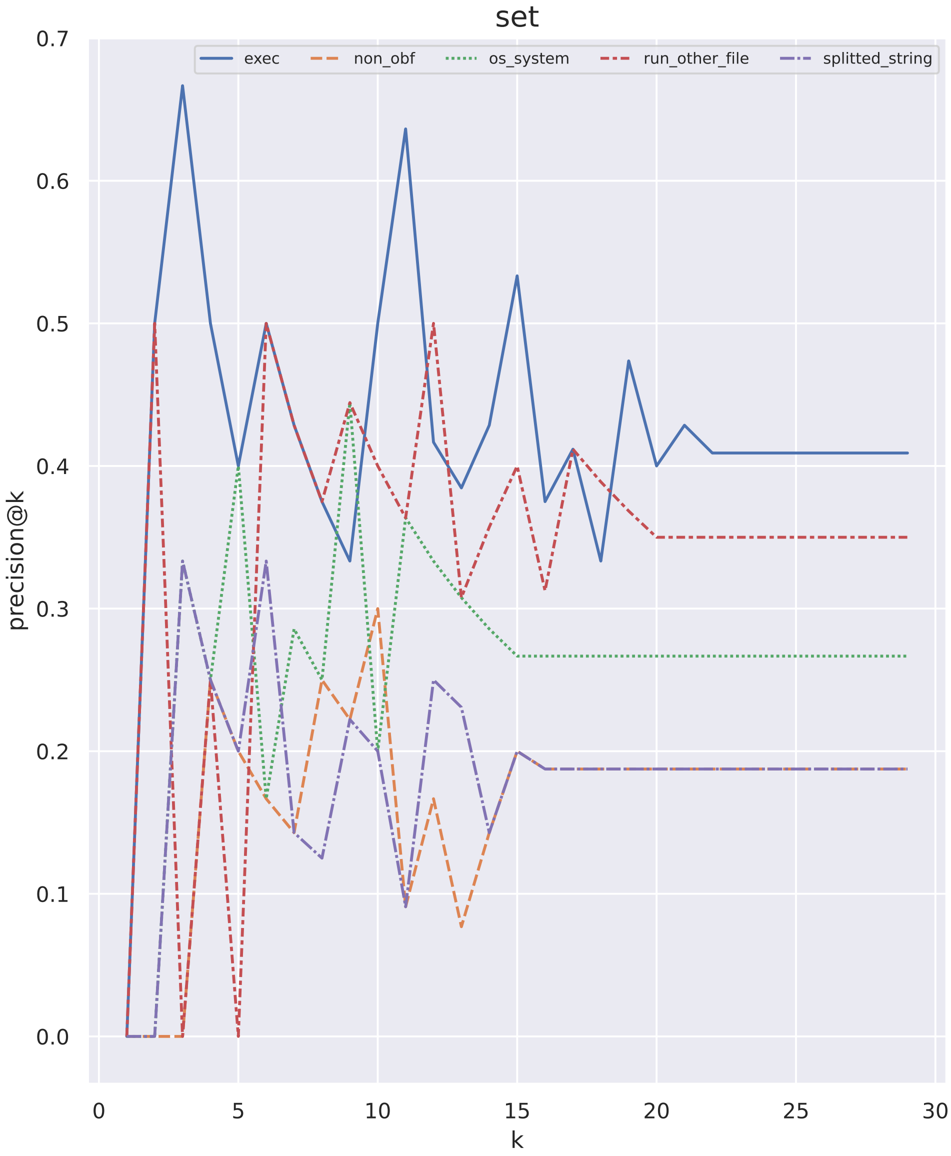

Figure S 7: The graphs below describe the *precision@k* results of the applied method in k in the range of 1 to 30. The presented results include all the *set* function implementations with different attacks (with the random code injection)

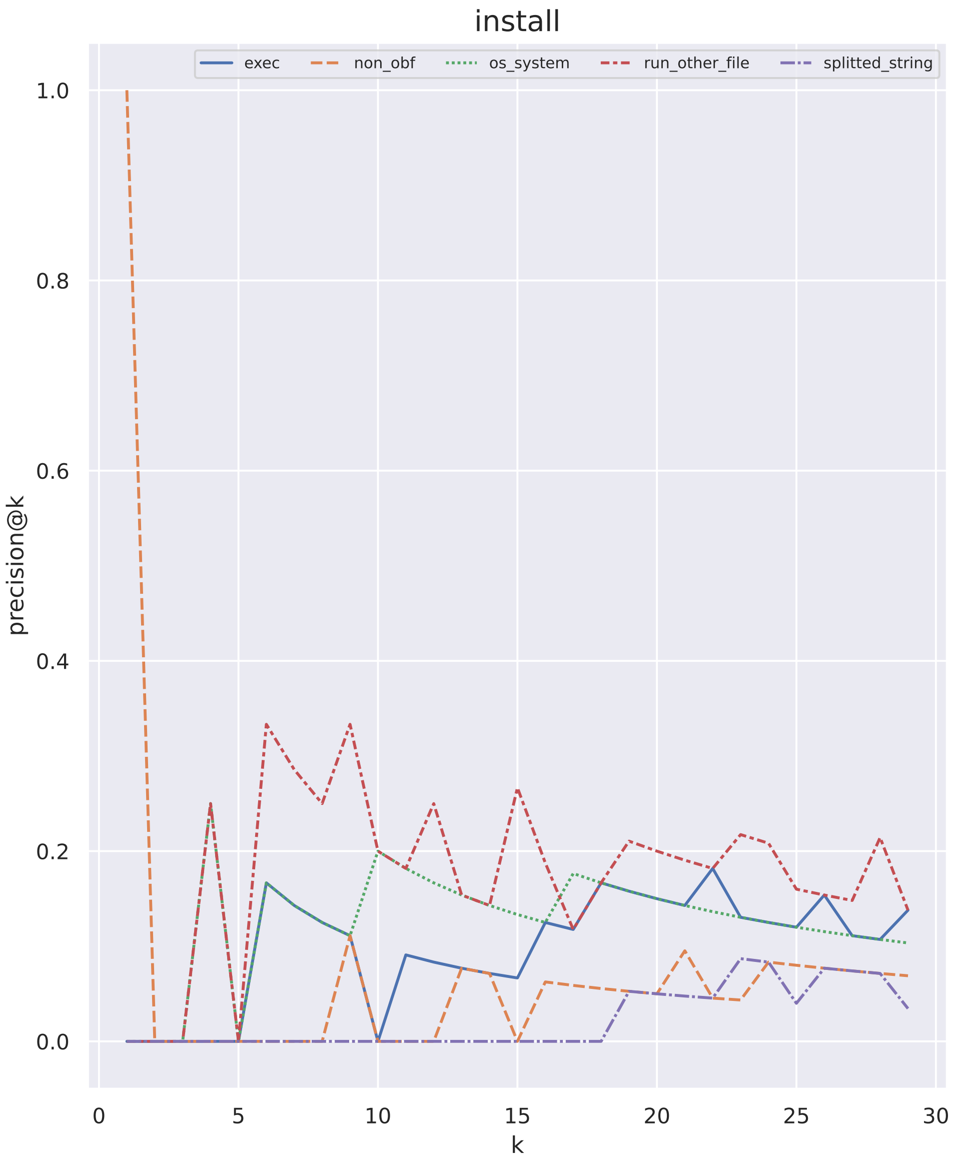

Figure S 8: The graphs below describe the *precision@k* results of the applied method in k in the range of 1 to 30. The presented results include all the *install* function implementations with different attacks (with the random code injection)
